# Viral piracy of host RNA phosphatase DUSP11 by avipoxviruses

**DOI:** 10.1371/journal.ppat.1013101

**Published:** 2025-04-21

**Authors:** Kayla H. Szymanik, Emily A. Rex, Vamshikrishna R. Pothireddy, Don B. Gammon, Dustin C. Hancks, Christopher S. Sullivan

**Affiliations:** 1 Department of Molecular Biosciences, The University of Texas at Austin, Austin, Texas, United States of America; 2 Department of Microbiology, UT. Southwestern Medical Center, Dallas, Texas, United States of America; 3 Department of Immunology, UT. Southwestern Medical Center, Dallas, Texas, United States of America; 4 Department of Molecular Biosciences, LaMontagne Center for Infectious Disease, The University of Texas at Austin, Austin, Texas, United States of America; University of California San Francisco, UNITED STATES OF AMERICA

## Abstract

Proper recognition of viral pathogens is an essential part of the innate immune response. A common viral replicative intermediate and chemical signal that cells use to identify pathogens is the presence of a triphosphorylated 5’ end (5’ppp) RNA, which activates the cytosolic RNA sensor RIG-I and initiates downstream antiviral signaling. While 5’pppRNA generated by viral RNA-dependent RNA polymerases (RdRps) can be a potent activator of the immune response, endogenous RNA polymerase III (RNAPIII) transcripts can retain the 5’ppp generated during transcription and induce a RIG-I-mediated immune response. We have previously shown that host RNA triphosphatase dual-specificity phosphatase 11 (DUSP11) can act on both host and viral RNAs, altering their levels and reducing their ability to induce RIG-I activation. Our previous work explored how experimentally altered DUSP11 activity can impact immune activation, prompting further exploration into natural contexts of altered DUSP11 activity. Here, we have identified viral DUSP11 homologs (vDUSP11s) present in some avipoxviruses. Consistent with the known functions of host DUSP11, we have shown that expression of vDUSP11s: 1) reduces levels of endogenous RNAPIII transcripts, 2) reduces a cell’s sensitivity to 5’pppRNA-mediated immune activation, and 3) restores virus infection defects seen in the absence of DUSP11. Our results identify a context where DUSP11 activity has been co-opted by viruses to alter RNA metabolism and influence the outcome of infection.

## Summary

Viruses face a critical challenge of disabling or avoiding host immune defenses. Cells typically recognize the presence of a virus through specific molecular markers, including triphosphorylated 5’ RNA ends that trigger immune responses. The host enzyme DUSP11 plays a key role in this process by modifying RNA and reducing immune activation. Such activity may prevent autoinflammation from host RNAs that, at least initially, start off 5’ triphosphorylated. However, so far, there are only limited contexts reported where DUSP11 activity matters during virus infection. We demonstrate here that an avipoxvirus lineage has pirated a DUSP11 gene from an ancestral host. These viral DUSP11 variants (vDUSP11s) can alter immunostimulatory host RNAs, counteract host defenses, and promote infection of a model virus. This research provides new insight into RNA biology during poxvirus infection, the overall function of viral and host DUSP11 enzymes, and more broadly how pathogens can manipulate molecular mechanisms to evade detection.

## Introduction

The interface between host defenses and viral antagonists is an evolutionary “arms race”, where both groups are under constant pressure to gain the advantage [1,2]. Host cells are armed with specialized pattern recognition receptors (PRRs) to sense conserved pathogen signatures, known as pathogen-associated molecular patterns (PAMPs), that initiate downstream antiviral signaling and protect against infection [3]. Viruses encode proteins to combat these pathways, and while they are limited by their coding capacity, they have a unique advantage due to their shorter generation times and faster mutation rates. This leaves host cells chasing a moving target, challenging both the specificity and selectivity of interactions.

RIG-I is a cytosolic PRR best characterized to detect structured or double-stranded RNA (dsRNA) bearing a di/tri-phosphate on the 5’-end, a common viral replicative intermediate generated by viral RNA-dependent RNA polymerases (RdRPs) [4–6]. Cellular RNAs are initially generated with a 5’-triphosphate end, and transcription via RNAP III occurs without a co-transcriptional capping mechanism [7]. In addition, recent studies have shown that RNAs generated by RNAP III can function as endogenous damage-associated molecular patterns (DAMPs) and activate RIG-I during virus infection [8–10]. This necessitates proper regulation of these signals to prevent immune activation in the absence of a pathogen.

DUSP11 is an RNA 5’-triphosphatase that converts 5’-di or triphosphorylated RNAs into their monophosphorylated form and can act on both host and viral RNAs [11–15]. DUSP11 activity on RNAs reduces their immunogenicity but also renders them more susceptible to nuclease decay [13,14]. Current literature details varying relationships between DUSP11 and virus infection. In the case of Hepatitis C virus (HCV), 5’-triphosphorylated transcripts are converted to monophosphates by DUSP11, rendering them susceptible to attack by endogenous exoribonucleases such as XRNs [13,16]. For bovine leukemia virus (BLV) and adenoviruses, viral miRNA processing is influenced by the presence of DUSP11, featuring a shift in the ratio of 5p:3p miRNA abundance in the absence of DUSP11 [12]. DUSP11 has been shown to modulate numerous host RNAP III transcripts, such as Y RNAs, SINEs, and vault RNAs [12]. We have previously demonstrated that DUSP11 activity renders cells and mice less active in inducing a RIG-I-mediated immune response [14]. These studies demonstrate that DUSP11 activity can be either pro-viral or anti-viral depending on the context, yet so far, there are few natural contexts identified in which DUSP11 activity levels are altered during infection [8,9].

Viruses encode diverse proteins targeting components of the host’s immune response to escape immune detection. Some viruses incorporate host genes into their genomes throughout their evolutionary history, and a large fraction of these “pirated” genes are involved in evading host antiviral defenses [3,17]. Here, we have identified the presence of DUSP11 homologs (vDUSP11) in some avipoxviruses (APVs) and unclassified APV-related viruses. Previous studies have shown that while poxviruses are DNA viruses, their infection can activate RIG-I [3,18]. Due to the established role of DUSP11 in modulating the innate immune response, we hypothesize that these viruses have acquired vDUSP11 with conserved catalytic activity to aid in immune evasion.

## Results

### DUSP11 (vDUSP11) genes are encoded by a subset of avipox and unclassified avipox-related poxvirus genomes

**Fig 1:**
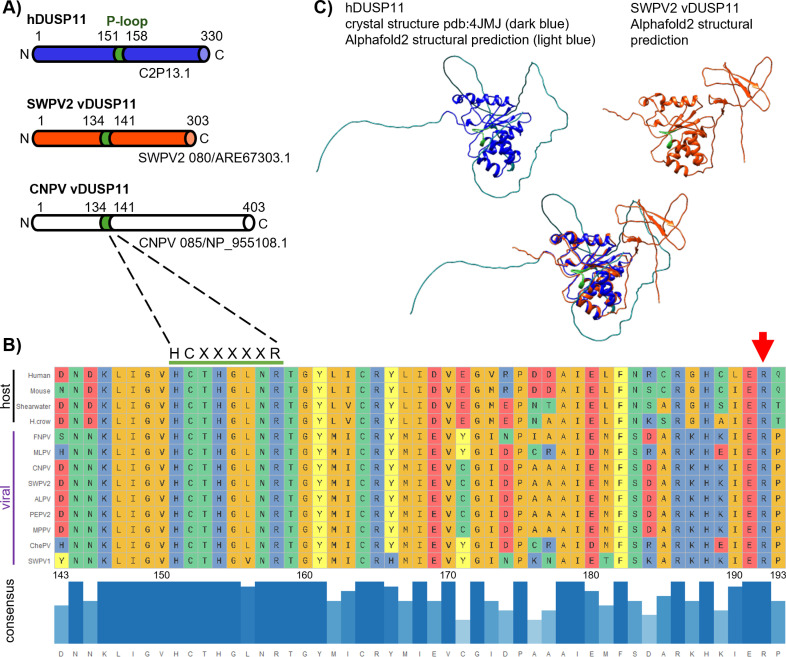
Avipoxviruses and related unclassified avipox-related poxviruses encode putative homologs of DUSP11. (A) Schematic of human *DUSP11* (hDUSP11) and select putative viral *DUSP11s* with predicted domains indicated. The shared colored domain (green) indicates hDUSP11 P-loop with corresponding identical sequences present in putative viral DUSP11 homologs (vDUSP11). Putative vDUSP11 includes shearwaterpox virus 2 vDUSP11 (SWPV2 vDUSP11) and canarypox virus vDUSP11 (CNPV vDUSP11). (B) Key catalytic residues are conserved between hDUSP11 and putative avipox vDUSP11. Multiple sequence comparison (Clustal Omega) of amino acid (AA) sequences for human DUSP11, mouse DUSP11, *C. borealis* (Shearwater), and putative vDUSP11s from shearwaterpox virus 1 (SWPV1), finchpox virus (FNPV), mudlarkpox virus (MLPV), cheloniidpox virus (ChePV), canarypox virus (CNPV), penguinpox virus 2 (PEPV2), albatrosspox virus (ALPV), magpiepox virus (MPPV), and shearwaterpox virus 2 (SWPV2). A subset of AAs surrounding the hDUSP11 P-loop are shown. Amino acids are colored based on side-chain chemistry. Bar graph indicating relative sequence conservation with consensus amino acid sequence determined from listed sequences. Taller bar height and darker blue color correspond to relative correlation to consensus sequence. The numbering below the consensus sequence corresponds to hDUSP11 amino acid numbering. hDUSP11 residue R192 is indicated by a red arrow. (C) Partial crystal structure of hDUSP11 (PDB:4JMJ, dark blue) with unsolved regions predicted using Alphafold2 (light blue) and Alphafold2 structural prediction shearwaterpox virus 2 vDUSP11 (orange) with structural overlay. Key catalytic residues (P-loop) present in hDUSP11 are indicated in green, and corresponding residues are also indicated on the putative SWPV2 vDUSP11 structural prediction.

DUSP11 is a member of the dual specificity phosphatase subgroup of type-I based cysteine-based protein tyrosine phosphatases (PTPs). Most members of the DUSP subgroup are active on protein substrates, but DUSP11 is atypical in that it is more active on RNA substrates [11]. Human DUSP11 (hDUSP11) (Chr2P13.1, O75319) has three isoforms, the major isoform being 330 amino acids (AA) in length. All DUSPs share a highly conserved phosphate-binding loop (P-loop) containing the consensus phosphatase sequence (HCXXXXXR; AA: 151–158 in human) [19] (Fig 1A). In addition, DUSP11 contains an R residue (R192 in hDUSP11), which has been previously suggested to be involved in the β-phosphatase activity, unique to RNA 5’-triphosphatases and lacking in mRNA capping enzymes [20]. Backed by recent insight into the immunomodulatory abilities of DUSP11, we hypothesized viruses might co-opt this activity. To this end, we set out to determine whether vertebrate virus genomes contain genes with similar features to DUSP11.

Using BLASTp analysis [21], we identified several APVs and avipox-related viruses predicted to encode proteins featuring high sequence identity (~44–60%) to human (hDUSP11) (S1 Table). Of those viruses, we initially sought to evaluate the putative viral homolog from one of the better-studied APVs, canarypox virus (CNPV), which encodes a 403 AA predicted homolog to DUSP11 (CNPV085/NP_955108.1) [44% (96/217) amino acid identity, (140/217) 64% positives (which indicates amino acids with similar chemical properties)] (Fig 1A). Canarypox viruses have been studied largely for use as a recombinant vaccine vector [22,23]. However, due to initial difficulties cloning the putative CNPV viral homolog, we pursued the closely related virus shearwaterpox virus 2 (SWPV2) as the model putative viral DUSP11 homolog. The SWPV2 genome contains a 303 AA predicted ORF (SWPV080/ARE67303.1) featuring high sequence identity to hDUSP11 [50% (94/188) amino acid identity, 67% (121/188) positives] (Fig 1A), hereafter vDUSP11. Notably, this protein (and other vDUSP11s) includes the R residue (AA: 161) corresponding to R192 of hDUSP11 (Fig 1B), suggesting a distinct role from existing mRNA capping enzymes. Using AlphaFold2 [24], we generated a structural prediction of the SWPV2 putative vDUSP11 homolog and aligned it to a partial crystal structure of hDUSP11 (PDB: 4JMJ; AA: 28–208) [25] with unsolved N-terminal and C-terminal extensions determined using AlphaFold2 (Figs 1C and S1). This structural analysis highlights that hDUSP11 and SWPV2 vDUSP11 are nearly superimposable, excluding the N- and C-terminal extensions, and that key catalytic residues (shown in green) are structurally conserved.

Using the SWPV2 vDUSP11 AA sequence as a query returns numerous host DUSP11 sequences, but not other phosphatases from related viruses, such as vaccinia VH1 (S2 Table). This reciprocal BLASTp analysis indicates that the progenitor of SWPV2 vDUSP11 was likely acquired by horizontal gene transfer (HGT) from a host copy of DUSP11. Many of the avipoxviruses that encode vDUSP11 infect hosts within the order of *Passeriformes*, making them a potential source of DUSP11. Further searches detected a total of 13* DUSP11 ORFs encoded by other APV/APV-related viruses, 2 of which are notably smaller (S2 and S3 Figs and S3 Table). (*Note: Our original studies identified 11 vDUSP11 homologs which are analyzed in the figures presented here. Two additional vDUSP11s were identified during database searches during the revisions and added to S3 Table). These findings show that multiple APV/APV-related viruses encode proteins that resemble DUSP11.

**Fig 2:**
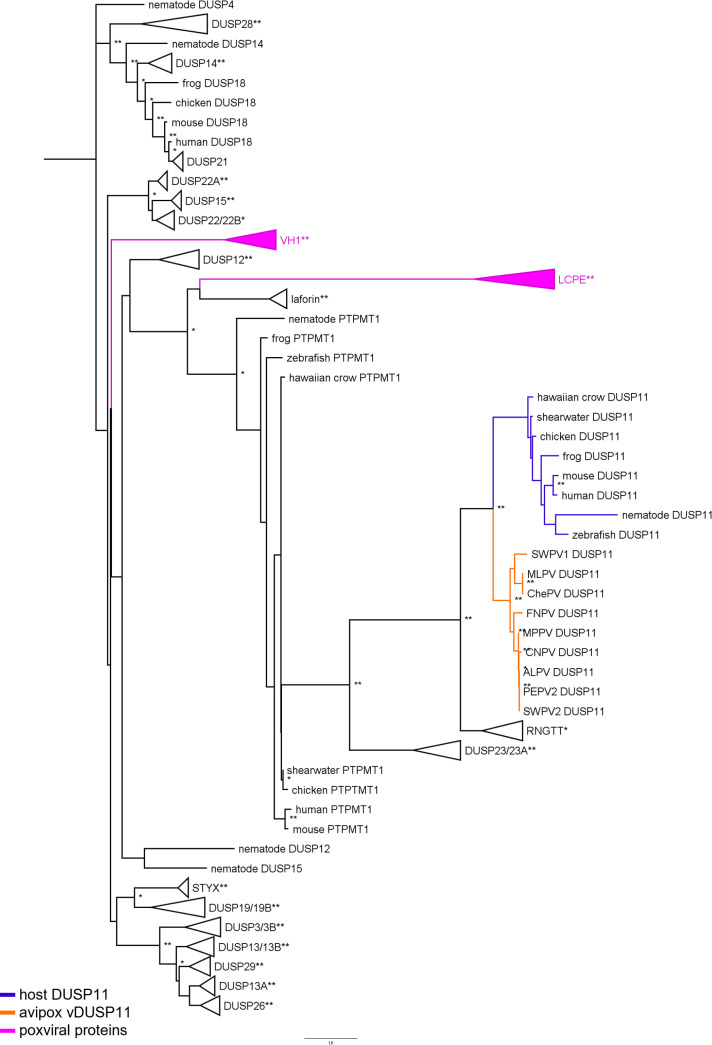
Phylogenetic analysis supports putative vDUSP11 acquisition from host DUSP11 sequence. An inferred tree built using 160 DUSP amino acid (AA) sequences by maximum-likelihood analysis using PhyML [42]. AA sequences for host atypical DUSPs, poxviral putative vDUSP11s, vaccinia virus DUSP *H1L* protein phosphatase and avipox homologs, and the large subunit the capping enzyme from poxviruses were aligned using Clustal Omega (S9 Fig) [46]. Clustal alignment was used to run PhyML analysis with LG + R model selected by SMS [53]. Sequences were retrieved from the HGNC sequence database, NCBI sequence database [41] and Uniprot (www.uniprot.org/) (S4 Table). Putative APV/AdjPV vDUSP11s (orange) cluster with host DUSP11s (blue) and not with other host protein DUSPs or other poxviral proteins (pink). Collapsed branches are used to represent proteins with homology that cluster together across numerous species. 100 bootstrap replicates were performed; branch support ≥50% (*) or ≥ 70% (**) are indicated. Nematode DUSP4, a member of the DUSP-family but not an atypical DUSP, was specified as the outgroup.

To further examine the acquisition of the putative vDUSP11 in an evolutionary context, we generated an inferred phylogenetic tree using AA sequences from proteins classified as atypical DUSPs from various hosts, the putative avipox viral DUSP11 sequences, poxviral VH1-like DUSPs from vaccinia virus (VACV) and corresponding avipoxvirus homologs, and the avipoxviral large capping enzyme subunit (S4 Table). The putative avipox vDUSP11s cluster with related host DUSP11s, and not with the other poxviral genes (Fig 2). These results further support the avipox vDUSP11 arose from host DUSP11 and not other viral or host DUSPs.

### vDUSP11s are enzymatically active

Based on AA sequence and predicted structural similarity, we hypothesized that these vDUSP11s possess similar catalytic activities as hDUSP11. hDUSP11 has been demonstrated to be active on 5’-triphosphate RNA, sequentially liberating both the γ- and β- phosphates, leaving a 5’-monophosphate [26]. This modification renders RNA susceptible to decay via monophosphate-dependent 5’-3’ exoribonuclease XRN1. We hypothesized that vDUSP11s are catalytically active RNA 5’-triphosphatases capable of performing similar functions as hDUSP11.

**Fig 3:**
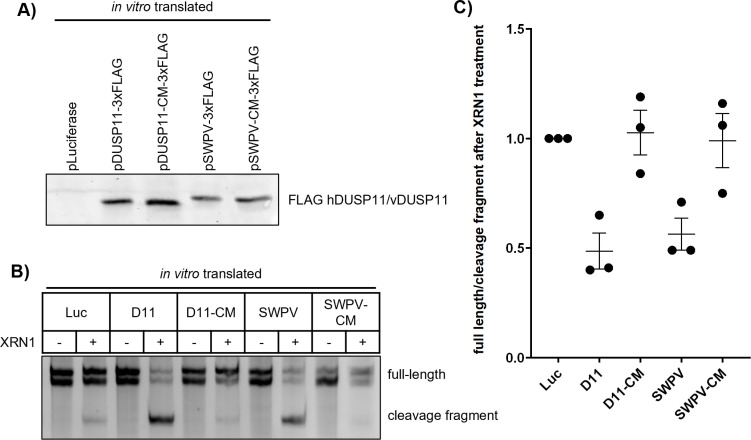
vDUSP11 sensitizes HCV 5’ UTR RNA to XRN-mediated degradation. (A) Confirmation of *in vitro* translated constructs via immunoblot analysis. Membrane was probed for FLAG-tagged proteins. Plasmid encoding luciferase was used for negative control reactions. (B) *In vitro* XRN susceptibility assay. *In vitro* transcribed HCV 5’ UTR RNA was incubated with *in vitro* translated products from (A) luciferase (Luc), hDUSP11 (D11), hDUSP11 catalytic mutant (D11-CM), shearwaterpox virus 2 vDUSP11 (SWPV) or shearwaterpox virus 2 vDUSP11 catalytic mutant (SWPV-CM)) and RNA was purified. Purified RNA was then subjected to treatment ± recombinant XRN1. Products were separated using urea PAGE and stained with EtBr. Migration of full-length HCV 5’ UTR is indicated, which for unknown reasons migrates as a doublet, the position of the faster-migrating cleavage fragment is indicated. (C) Graphical representation of (B), displaying ratio of band intensity of the ratio of HCV 5’ UTR full-length doublet bands to cleavage fragment band from the XRN-treated to the -XRN1 treatment reactions. Values are represented relative to the negative control Luc treatment. Data are derived from n = 3 independent replicates. In all panels, data are represented as mean ± SEM.

To assess this hypothesis, we applied a previously established assay to measure the ability of vDUSP11 to sensitize *in vitro* transcribed RNAs to decay via XRN1 [27,28]. 5’-RNA phosphatase treatment followed by XRN treatment of a hepatitis C virus (HCV) 5’-UTR RNA results in the generation of a stable quantifiable smaller fragment [13,27,28]. We generated *in vitro* transcribed and translated flag-tagged full-length proteins for hDUSP11, negative control hDUSP11, catalytically inactive mutant (D11-CM) (C152S), the SWPV2 vDUSP11, and the predicted SWPV2 vDUSP11 catalytically inactive mutant (SWPV-CM) (C135S). We also included an additional negative control of untagged luciferase. Protein expression was confirmed via immunoblot analysis (Fig 3A). We treated *in vitro* transcribed HCV 5’ UTR RNA with the *in vitro* transcribed/translated constructs. Following phosphatase treatment, we purified the RNA and then treated ± recombinant XRN1. We evaluated the abundance and length of RNA by using a urea-PAGE gel stained with ethidium bromide (EtBr) to visualize RNA. All conditions using treatment with XRN1 resulted in a small degree of generation of a shorter RNA fragment, suggesting low levels of susceptible monophosphate RNA present in the substrate population (Fig 3B). However, treatment with catalytically active hDUSP11 or the SWPV2 vDUSP11, but not either of the catalytic mutants (CM), resulted in a substantial reduction in the full-length HCV 5’ UTR fragment. Quantification indicated a ~ 30–50% increase in the ratio of degradation fragment to full-length RNA seen following XRN1 treatment (Fig 3B and 3C). To confirm that vDUSP11 directly provides the relevant enzymatic activity assayed here, we performed this same reaction using purified chimeric GST fusion protein containing the amino terminus of SWPV vDUSP11 encompassing the catalytic core (amino acids 1–197). This protein showed activity while the CM (C135S) negative control GST fusion protein did not (S4 Fig). These data demonstrate that vDUSP11, in the absence of any other stoichiometric host proteins, is an RNA triphosphatase. This result is what is expected for the known catalytic activity of DUSP11 to act on 5’-triphosphorylated RNA, sensitizing them to decay via XRN1. Thus, SWPV2 vDUSP11 is a catalytically active 5’-RNA triphosphatase and this activity depends on cysteine in the catalytic pocket, as previously demonstrated for hDUSP11.

### vDUSP11s modulate sensitivity to immune activation triggered by 5’-triphosphate RNAs

We next determined if vDUSP11 shares additional functions with hDUSP11. RIG-I is a key PRR that recognizes structured or double-stranded 5’ di-/tri-phosphorylated RNAs, which, when activated, leads to the induction of antiviral signaling, including transcripts such as ISG15 and IFN-β [4–6]. We hypothesized that APVs/APV-related viruses have acquired vDUSP11 to mimic host DUSP11’s immunomodulatory properties, which alter the sensitivity of RIG-I activation and modulate RNAP III transcripts upregulated in the context of various virus infections [8,9,14].

**Fig 4:**
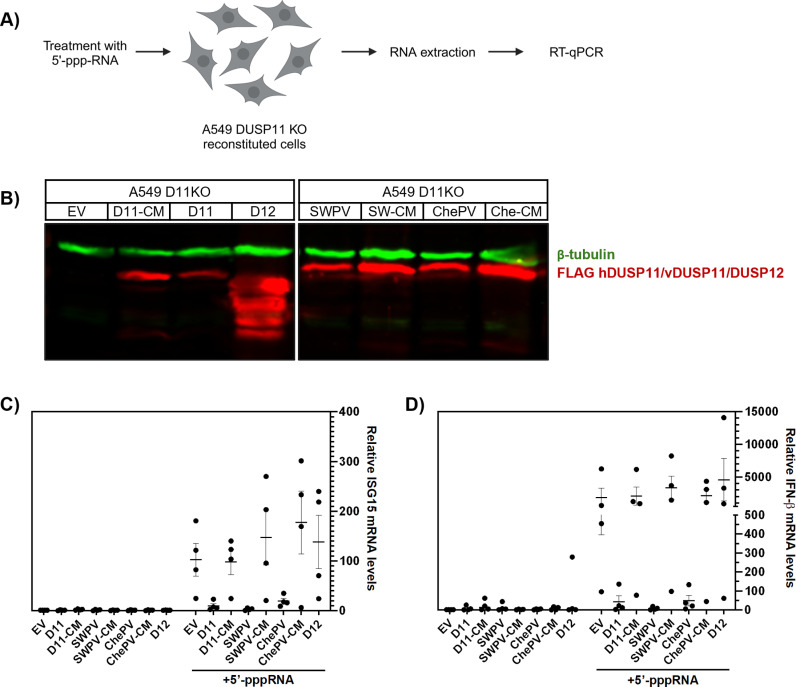
vDUSP11 modulates immune activation in response to liposomal 5’-ppp-RNAs. (A) Schematic diagram of the 5’-triphosphate RNA (5’-ppp-RNA) transfection assay. A549 DUSP11 knockout (KO) reconstituted cells (12-well) were transfected with 5–10 ng of *in vitro* transcribed 5’-ppp-RNA for 18 hours followed by RT-qPCR to assay induction of ISGs. (B) Immunoblot analysis of A549 DUSP11 knockout (KO) cells transduced with pLenti empty vector (EV), pLenti hDUSP11-3xFLAG (D11), pLenti hDUSP11 catalytic mutant-3xFLAG (D11-CM), pLenti shearwaterpox virus 2 vDUSP11-3xFLAG (SWPV), pLenti shearwaterpox virus 2 vDUSP11 catalytic mutant-3xFLAG (SW-CM/SWPV-CM), pLenti cheloniidpox virus 1 vDUSP11-3xFLAG (ChePV), pLenti 3xFLAG-cheloniidpox virus 1 vDUSP11 catalytic mutant-3xFLAG (Che-CM/ChePV-CM), or negative control pLenti DUSP12-3xFLAG (D12). (C) RT-qPCR analysis of *ISG15* and (D) *IFNB1* mRNA levels in A549 DUSP11 KO reconstituted cells following transfection with either lipofectamine alone or 5’-ppp-RNA, normalized to *GAPDH* mRNA levels. Results are represented relative to those of empty vector expressing cells. Data are derived from n = 4 independent replicates and are represented as mean ± SEM. Schematic figures in Fig 4A were created with BioRender.com.

To assay the functions of the vDUSP11s in cell culture, we transfected immunostimulatory RNAs (Fig 4A) into previously described A549 DUSP11 knockout (KO) cells [12] stably expressing various DUSP11s and measured RIG-I activity via IFN-stimulated gene (ISG) expression. Included in this analysis is a second full-length vDUSP11 from cheloniidpox virus 1 (ChePV) [44% (88/201) amino acid identity, 66% (133/201) positives to hDUSP11]. (We selected vDUSP11 from ChePV due to this virus being isolated from a non-avian host). We generated cells stably reconstituted with a control empty-vector (EV) or the following N-terminally flag-tagged proteins: 1) wild-type hDUSP11 (D11), 2) hDUSP11 catalytic-mutant (C152S, D11-CM), 3) SWPV2 vDUSP11 (SWPV2), 4) SWPV2 catalytic-mutant vDUSP11 (C135S, SWPV2-CM), 5) ChePV vDUSP11 (ChePV), 6) ChePV catalytic-mutant of ChePV (C135S, ChePV-CM), or 7) phosphatase DUSP12 (D12) (a canonical DUSP known to be active on protein substrates) as a negative control protein. Protein expression for each was confirmed via immunoblot analysis (Fig 4B).

Upon transfection of 5’-triphosphate RNA, cells expressing hDUSP11, SWPV vDUSP11, or ChePV vDUSP11, but not the empty-vector nor any catalytic mutants/negative controls, displayed a reduction in both induced *ISG15* and *IFN-β* ISG transcript levels (Figs 4C and S5). Compared to empty vector cell lines, those containing either hDUSP11 or the SWPV and ChePV vDUSP11s showed on average a ~ 40–50-, ~ 40–90-, and ~ 30–50-percent reduction in induced *ISG15* mRNA levels, respectively, and all showed a greater than a ~ 70–90-percent average reduction in induced *IFNB1* mRNA levels. These results indicate that both vDUSP11s can modulate immune activation, as previously shown for hDUSP11.

### vDUSP11 catalytic activity promotes VSV virus replication

The role of DUSP11 in virus infection is context-dependent, however, previous work from our lab has shown that some viruses (e.g., + ssRNA sindbis virus (SINV) and -ssRNA vesicular stomatitis virus (VSV)) benefit from DUSP11 activity during infection [14]. Both SINV and VSV have been shown to produce 5’-triphosphorylated RNA as part of their replication cycle. Additionally, host RNAs have also been implicated in activating RIG-I upon infection with some RNA viruses [9]. We hypothesized that vDUSP11 can enhance virus infection in a manner similar to hDUSP11. To investigate this, we infected A549 DUSP11 KO cells reconstituted with the various viral and human DUSP11s with M51R mutant VSV for 48 hours at an MOI of 0.01 PFU/cell. VSV M51R lacks the ability to fully block host gene expression and, as a result, stimulates a stronger interferon response [29].

**Fig 5:**
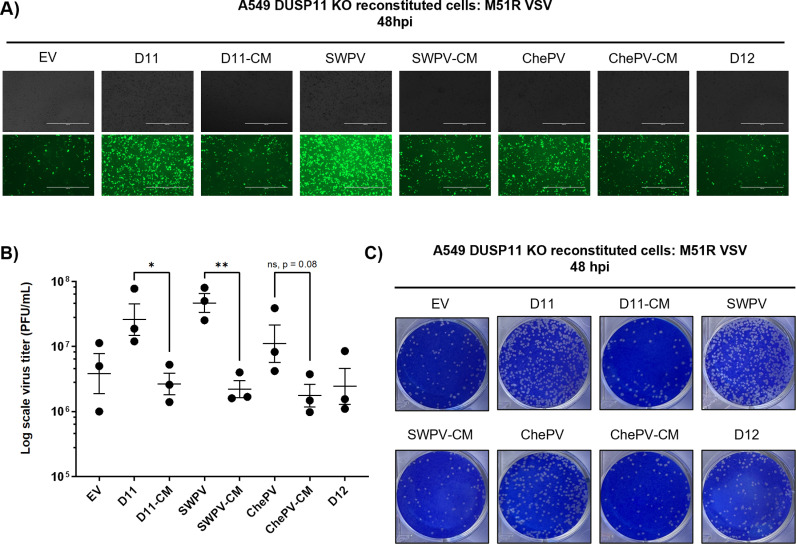
vDUSP11 catalytic activity promotes VSV virus replication. (A) Representative GFP images of A549 DUSP11 knockout (KO) cells stably reconstituted with either empty vector (EV) plasmid, hDUSP11 (D11), hDUSP11 catalytic mutant (D11-CM), shearwaterpox virus 2 vDUSP11 (SWPV), shearwaterpox virus 2 vDUSP11 catalytic mutant (SWPV-CM), cheloniidpox virus vDUSP11 (ChePV), cheloniidpox virus vDUSP11 catalytic mutant (ChePV-CM), or protein phosphatase DUSP12 (D12) after infection with GFP-M51R VSV at an MOI of 0.01 PFU/cell at 48 hours post-infection (hpi). (B) VSV viral titer of indicated A549 DUSP11 KO reconstituted cells determined by plaque assay analysis at 48 hpi. (C) Representative images of plaque assay analysis of virus supernatant at 48 hpi. Data are derived from n = 3 independent replicates. Data are presented as mean ± SEM. (*) *P *< 0.05; (**) *P *< 0.01 (two-tailed Student’s *t*-tes*t*).

Cells expressing either hDUSP11, SWPV vDUSP11, or ChePV vDUSP11, but not catalytic mutants or negative controls (D12/EV) enhanced VSV infection (Fig 5A-C). Compared to cells expressing only the empty vector, those reconstituted with hDUSP11 had, on average, a ~ 7-fold increase in the total virus they produced, consistent with previously published findings [14]. Cells expressing the SWPV vDUSP11 had, on average, a ~ 20-fold increase in viral titer, and those expressing the ChePV vDUSP11 had a ~ 3-fold increase in viral titer. These findings lend further support to evolutionarily conserved shared functionality between host and viral DUSP11s.

### vDUSP11 reduce immune activation in response to vaccinia (VACV) poxvirus infection

Given that the presence of vDUSP11 can alter the outcome of virus infection for RNA viruses as previously demonstrated for hDUSP11, we next sought to evaluate these vDUSP11 in a context closer to natural infection. A lack of a facile model system limited our ability to test these proteins during avipoxvirus infection, so we instead utilized the well-studied, related poxvirus: VACV. While VACV does not appear to have acquired a host copy of DUSP11, studies have reported interactions between the VACV protein E3 and RIG-I. E3, encoded by the gene *E3L*, has been reported to have many functions on the interface of pathogen-host interactions [30,31]. While a role for E3 in altering RIG-I activation has been proposed [30,32,33], the extent of this in altering immune signaling is unclear.

**Fig 6:**
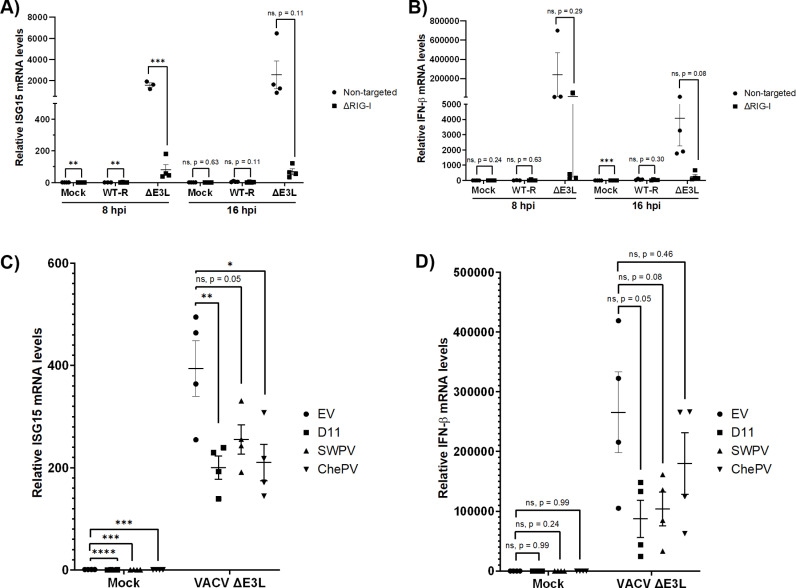
DUSP11 reduces immune signaling in response to infection by VACV ∆*E3L.* Previously characterized A549^NT^ (non-targeted) and A549 RIG-I knock out cells (∆RIG-I) [34] were either mock infected (Mock), infected with wild-type recombinant vaccinia virus (VACV) (WT-R), or infected with an *E3L* deficient VACV (∆*E3L*/VACV ∆*E3L*). (A). RT-qPCR analysis of *ISG15* and (B) *IFNB1* mRNA normalized to *GAPDH* mRNA levels. Results are represented relative to those of non-targeted cells. (C) RT-qPCR analysis of *ISG15* and (D) *IFNB1* mRNA normalized to *GAPDH* mRNA of A549 DUSP11 KO cells expressing either empty vector (EV), hDUSP11 (D11), shearwaterpox virus vDUSP11 (SWPV), or cheloniidpox virus vDUSP11 (ChePV) at 16 hpi following either mock infection or infection with VACV ∆*E3L*. Results are represented relative to mock infected A549 DUSP11 KO cells reconstituted with EV. For panels A and B, non-targeted cells infected with either WT-R or VACV ∆*E3L* at 8 hpi, data are derived from n = 3 independent replicates. For all other time points, data are derived from n = 4 independent replicates. All data are represented as mean ± SEM. (*) *P *< 0.05; (**) *P *< 0.01; (***) *P *< 0.001; (****); *P *< 0.0001. Statistical analysis for panels A and B included two-tailed students t-tests; C and D included one-way ANOVAs followed by Dunnett’s multiple comparison test.

We first set out to determine if there were any detectible differences in infection or immune signaling in response to infection by VACV *ΔE3L* dependent on the presence of RIG-I. We infected previously characterized A549 RIG-I knockout (ΔRIG-I) and non-targeted (NT) control cells [34] with either a recombinant WT (WT-R) or an *E3L* deletion mutant of VACV (VACV *ΔE3L*) and quantified viral titers and mRNA levels of *ISG15* and *IFN-β* at 8 and 16 hours post infection (hpi). Viral titers measured at 16 hpi were unaffected by the presence of RIG-I (S6A-D Fig). Infection with WT-R VACV resulted in low levels of *ISG15* and *IFN-β* transcripts in both cell lines, with on average a 0.5 to 3-fold increase in either transcript detected in the presence of RIG-I compared to in the ΔRIG-I cells (Fig 6A and 6B). However, infection with VACV *ΔE3L* resulted in a 20–52-fold increase of either *ISG15* or *IFN-β* mRNA levels detected in the presence of RIG-I compared to in the ΔRIG-I cells. These data compellingly support that E3 can function to reduce RIG-I-dependent immune activation during VACV infection.

Next, we evaluated if the presence of either hDUSP11 or vDUSP11 impacted immune signaling in response to infection by VACV *ΔE3L*. Overall, we observed only minor differences in virus production between cell lines at 16 hours post infection (hpi) (S6E-G Fig). We quantified *ISG15* and *IFN-β* mRNA levels at 16 hpi from cells either mock-infected or infected with VACV *ΔE3L* (Fig 6C and 6D). In most cases in uninfected states, we observed that cells expressing only the empty vector (EV) showed a slight increase in expression of either *ISG15* or *IFN-β* mRNA under mock infection conditions, consistent with an increased propensity for immune activation in the absence of DUSP11. In response to VACV *ΔE3L* infection, we observed that cells expressing hDUSP11 (D11) had on average a 2-fold reduction in *ISG15* mRNA levels and 3.6-fold reduction in *IFN-β* mRNA levels compared to the EV expressing cell line. Expression of either SWPV2 vDUSP11 (SWPV) or ChePV vDUSP11 (ChePV) resulted in an on average 1.5-fold and 1.9-fold reduction in *ISG15* mRNA levels, respectively, and an average 2.7-fold reduction and 1.5 in *IFN-β* mRNA levels, respectively (Fig 6C and 6D). These data demonstrate that expression of either hDUSP11 or vDUSP11 results in modest reduced immune signaling in response to infection by VACV *ΔE3L*. These data further support a model whereby vDUSP11s reduce RIG-I-dependent immune activation during avipoxvirus infection.

### vDUSP11s alter steady-state levels of select RNA polymerase III RNAs

Previous work from our lab demonstrated that DUSP11 modulates the 5’-triphosphate status and steady-state levels of several RNAP III transcripts, including vault RNAs (vtRNAs), Y RNAs, and RMRP [12]. This activity could be advantageous to viruses as RNAP III transcripts can activate host PRRs such as RIG-I during virus infection [8,9]. Based on this, we sought to determine if the presence of vDUSP11 can impact the steady-state levels of endogenous RNAP III transcripts.

**Fig 7:**
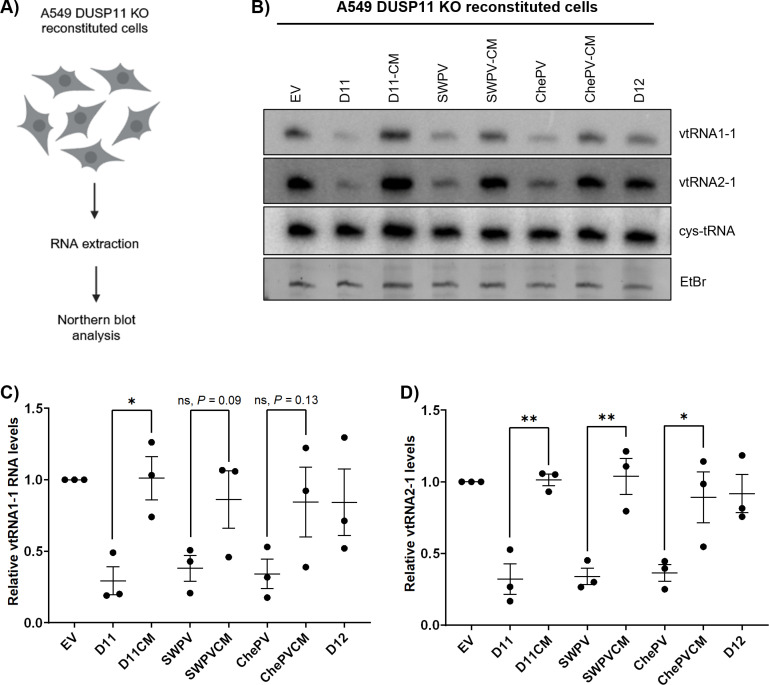
vDUSP11 modulates steady-state RNA levels of endogenous RNAP III transcripts. (A) Schematic diagram of vtRNA northern blot. RNA was collected from resting A549 DUSP11 KO cells stably reconstituted with empty vector (EV) plasmid, hDUSP11 (D11), hDUSP11 catalytic mutant (D11-CM), shearwaterpox virus 2 vDUSP11 (SWPV), shearwaterpox virus 2 vDUSP11 catalytic mutant (SWPV-CM), cheloniidpox virus vDUSP11 (ChePV), cheloniidpox virus vDUSP11 catalytic mutant (ChePV-CM), or negative control protein phosphatase DUSP12 (D12). Purified RNA was then subjected to northern blot analysis. (B) Northern blot analysis of vtRNA1–1 and vtRNA2–1 using RNA from A549 DUSP11 KO reconstituted cells. (C) Graphical representation of relative band intensity of vtRNA1–1 and (D) vtRNA2–1 normalized to the relative band intensity of the 5’ monophosphate control cysteine-tRNA. Values are represented relative to the A549 DUSP11 KO + EV cell line. Data are derived from n = 3 independent replicates. In all panels, data are represented as mean ± SEM. (*) *P *< 0.05; (**) *P *< 0.01 (two-tailed Student’s *t*-tes*t*). Schematic figures in Fig 7A were created with BioRender.com.

To evaluate if vDUSP11 catalytic activity can alter the abundance of endogenous RNAPIII transcripts, we harvested total RNA from A549 DUSP11 KO reconstituted cells and conducted northern blot analysis (Fig 7A). We probed for vtRNA1–1 and vtRNA2–1, using the 5’ monophosphate tRNA cysteine (tRNA-Cys) as a loading control. In the presence of either hDUSP11 or either vDUSP11, we observed ~30–70% reduced levels of vtRNA1–1 and vtRNA2–1 when compared to the empty vector negative control (Figs 7B–7D and S7). These data suggest that vDUSP11s can alter vtRNA levels comparable to hDUSP11.

### vDUSP11s exhibit pan-cellular localization

RIG-I is predominantly localized to the cytoplasm [35], but RNAP III RNAs are typically distributed in both the cytoplasm and nucleus. In resting cells, hDUSP11 has been shown to predominantly reside in the nucleus [36]. However, in infected cells, DUSP11 must also be active in the cytoplasm, as both HCV and VSV transcripts are direct substrates that are restricted to the cytosol [13,14]. Poxviruses are DNA viruses, but unlike the majority of DNA viruses, they replicate in the cytoplasm. We sought to determine how vDUSP11 localization compares to hDUSP11.

**Fig 8:**
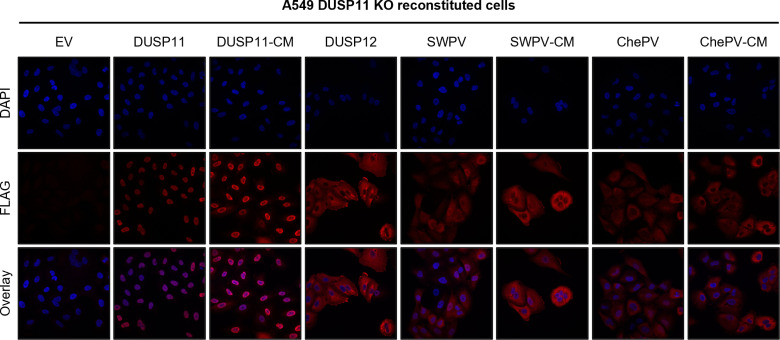
vDUSP11s differ in subcellular localization compared to hDUSP11. Representative confocal images of A549 DUSP11 KO cells stably expressing either an empty vector (EV) plasmid or individual 3xFLAG tagged proteins including hDUSP11 (D11), hDUSP11 catalytic mutant (D11-CM), SWPV2 vDUSP11 (SWPV), SWPV2-CM (SWPV-CM), ChePV1 vDUSP11 (ChePV), ChePV1 vDUSP11-CM (ChePV-CM), or negative control protein phosphatase DUSP12 (D12). Prolong Gold Antifade Mountant (Thermo Fisher Scientific) was used in slide preparation to visualize nuclei.

We performed confocal immunofluorescence microscopy using the various hDUSP11 and vDUSP11 reconstituted A549 DUSP11 KO cell lines. As expected, resting cells expressing hDUSP11 and hDUSP11-CM displayed predominantly nuclear localization (Fig 8). In contrast, both vDUSP11s and their catalytic mutants appear to be pan-cellular with substantial abundance in the cytoplasm (Fig 8). The localization of these vDUSP11s is consistent with overlapping the site of poxviral replication and replicative intermediates.

### Synteny analysis supports a single avipox vDUSP11 acquisition event from an ancestral host

Viral piracy of host proteins often occurs when genes have important immunological functions [2]. Due to their role at the host-pathogen interface, pirated genes are often subject to volatile evolution, which can include frequent duplications and loss [2,37]. For poxviruses in particular, genes that inhibit host defense often cluster together within the genome and often appear in regions towards the ends of the genome [38–40]. Comparing the genomic localization of these vDUSP11s can also inform the evolutionary history of vDUSP11 including whether these genes were acquired from the host multiple times or through a single event.

**Fig 9:**
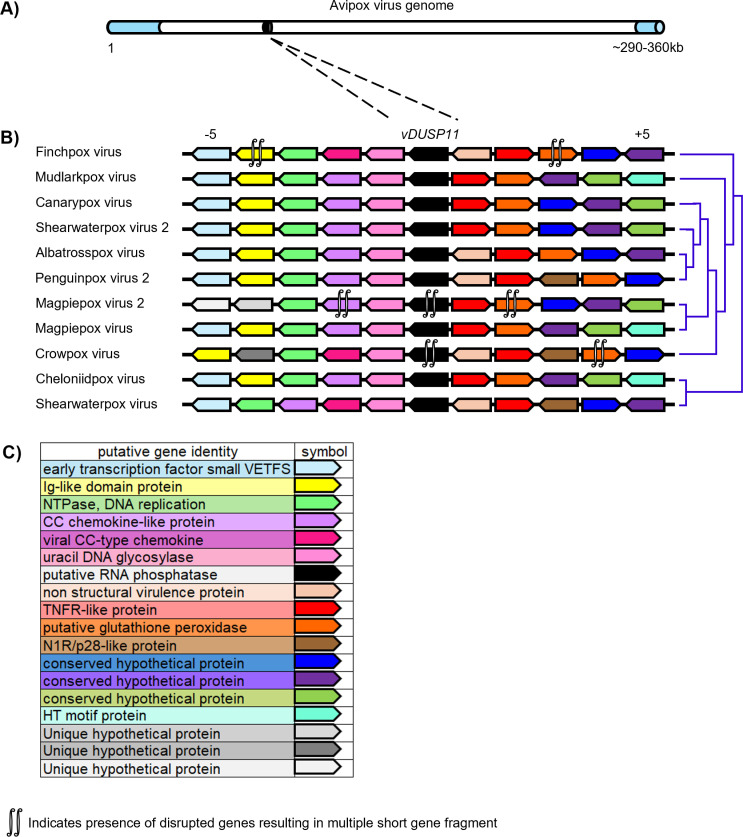
A single acquisition event for vDUSP11 is indicated by synteny analysis. (A) Graphical representation of avipoxvirus genome using published information from the canarypox virus genome as a model [62]. Inverted terminal repeats (ITRs) are indicated in light blue, relative genome position of *vDUSP11* indicated in black. (B) Analysis of upstream and downstream flanking genes indicated *vDUSP11*s (black) reside at similar genomic locations across poxvirus genomes. Protein homologs were determined using reciprocal BLAST hits for the 5 genes upstream (left of *vDUSP11*) and 5 genes downstream (right of *vDUSP11*) of *vDUSP11*. Homologous genes are indicated by shared color. Genes oriented to the right indicate ORFs on the top strand while genes oriented to the left indicate genes on the bottom strand. Cladogram indicating virus relatedness from published data [63] to the right of each gene diagram. (C) Corresponding functional annotation for genes indicated in (B). Predicted functional annotations were determined using NCBI database genome annotations [41]. For genes lacking descriptive annotations, more detailed annotations based on identified homologous genes were used (S5 Table).

To assess the acquisition of vDUSP11 into the various APV/APV-related genomes, we analyzed the genomic location and adjacent genes. Using reference genome sequences in the NCBI database [41] for each APV/APV-related virus containing a vDUSP11, we analyzed genes upstream and downstream of the vDUSP11 coding region (Fig 9 and S5 Table). We found that while vDUSP11 is not encoded in the inverted terminal repeats (ITRs), in many cases, it is surrounded by other genes predicted to be involved in modulating the host response to infection (Fig 9 and S5 Table). Due to the similarity in the order of flanking genes, these data suggest that vDUSP11s are orthologous and likely acquired by a single acquisition event.

During this analysis, we noted that the vDUSP11 loci for crowpox virus and magpiepox virus 2 are substantially shorter. The coding region for the magpiepox virus 2 vDUSP11 encodes a predicted 60 AA protein retaining key residues essential for catalytic activity. However, it is unclear if this is sufficient for functionality. In the case of crowpox virus, comparing the genomic location to other closely related viruses showed a mutated start codon resulting in a protein not predicted to retain catalytic activity. The presence of these truncated forms is consistent with this locus being a site of evolutionary pressure. Thus, these evolutionary profiles are consistent with what would be expected if vDUSP11 activity is involved in modulating host defenses.

Our findings support the model of a single acquisition event of a host DUSP11 in an ancestor virus that gave rise to the modern lineage of avipox and avipox-related viruses with vDUSP11s. To decipher a potential source of acquisition, we performed reciprocal BLASTp analysis for each viral homolog to identify the most similar host DUSP11. Based on both BLASTp E-value and percent identity, we identified the most similar host DUSP11 to be from *Corvus hawaiiensis* (Hawaiian crow) to the penguinpox 2 vDUSP11 [53% (94/176) amino acid identity, 71% positives (126/176)] (S6 Table). Furthermore, this host is recalled for all the full-length vDUSP11, except for mudlarkpox virus for which *Varanus komodoensis*, (Komodo dragon) was recalled [51% (96/189) amino acid identity, 69% positives (131/189)] (S7 Table). This further supports the likelihood that the vDUSP11s were acquired from an avian species and potentially one closely related to *Corvus hawaiiensis.*

To further evaluate the acquisition source of APV/APV-related vDUSP11, we performed phylogenetic analysis using eight avipox vDUSP11 sequence and eighteen selected host DUSP11 AA sequences (S8 Table). Using PhyML [42] to generate an inferred phylogenetic tree, we found that the avipox vDUSP11 clusters with avian DUSP11 (S8 Fig). These data lend additional support for an avian origin for the various avipox vDUSP11. Combined, the above data demonstrate that APV/APV-related viruses have acquired a viral homolog of host RNA phosphatase DUSP11, which is enzymatically active and features characteristics of a protein involved in the modulation of the host immune response.

## Discussion

Historically, unraveling the mechanisms of how viruses replicate has allowed for a greater understanding of not only viruses but also of how cells function. In addition, viral piracy of host genes can implicate that the activity of a host factor directly influences infection outcomes [2]. A detailed understanding and characterization of viral replication strategies and immune pathways is essential to developing new therapies and applications. Our studies of vDUSP11s not only shed light on how APVs/APV-related viruses deal with barriers to viral replication, but also support the role of DUSP11 in modulating the antiviral response.

As previously published, knockout of cellular hDUSP11 leads to increased sensitivity to RIG-I-related immune activation [14]. Herein, we also find that SWPV2- and ChePV- encoded vDUSP11s can modulate immune activation in response to liposomal 5’-triphosphate RNAs (Fig 4). The functional relevance of vDUSP11 activity is illustrated by these factors being pro-viral in the context of VSV infection, as previously seen for hDUSP11 (Fig 5). These observations demonstrate similar functionalities of host and viral DUSP11 and are consistent with a role of vDUSP11s in modulating RIG-I during Avipoxvirus infection. Due to the current lack of a facile model system for these APVs/APV-related viruses, this model has not been tested under native infection.

However, we demonstrate that vDUSP11s can reduce RIG-I signaling in the context of poxvirus infection in cells infected with a well-studied mutant poxvirus known to instigate enhanced immune signaling: Heterologous expression of vDUSP11s can partially reduce RIG-I signaling in cells infected with VACV *ΔE3L* mutant poxvirus (Fig 6C and 6D). We note, in in this heterologous system, vDUSP11s have little effect on VACV virus replication (S6G and S6H Fig). Consistent with this, genetic ablation of RIG-I fails to rescue *ΔE3L* replication (S6D Fig), demonstrating that *E3L* must perform other functions in addition to preventing RIG-I activation. APV/APV-related viruses do not encode E3, and VACV and other closely related poxviruses do not encode vDUSP11. Given the extreme fitness defect of VACV *ΔE3L* [31], the absence of E3 in APVs likely necessitates the presence of additional proteins involved in immune modulation, such as vDUSP11.

It is established that for some viruses, viral RNA produced during infection can have immunostimulatory properties, in part, by activating host PRRs [4,6]. However, there is a growing pool of evidence demonstrating that endogenous RNAs may also be relevant activators of RIG-I during virus infection [8,9]. Here, we show that vDUSP11 can modulate steady-state levels of endogenous RNAP III transcripts (Fig 7). It remains a possibility that the ability of vDUSP11 to modulate RNAP III transcripts is not merely a “passenger” of, but rather a “driver” of, the relevant biological functions of these proteins to prevent RNAPIII transcripts from acting as DAMPs/PAMPs during APV/APV-related virus infection.

5’-triphosphate RNA can be a replicative intermediate of RNA virus replication, but the mechanism for how DNA virus infection enhances levels of total (host and viral) 5’-triphosphate RNA is less clear. Endogenous RNAP III transcripts are a potential RIG-I agonist [8–10], which could explain the source for APV/APV-related virus infection. Another proposed model for the source of 5’-triphosphate RNA during DNA virus infection is RNAP III translocation to the cytoplasm, which then generates immunostimulatory transcripts from foreign DNA templates [18,43]. These models are not mutually exclusive, and both suggest a role for host RNAP III in activating the immune response during poxvirus infection.

During virus infection, the site within the cell where viral replication occurs dictates the availability of cellular resources and which host defense factors are present. DNA viruses commonly replicate in the nucleus to allow access to host replication machinery. Atypically, poxviruses are large DNA viruses that replicate in the cytoplasm, as they have large genomes that encode their own replication machinery [44]. It follows that via immunofluorescence, we observed vDUSP11 to be pan-cellular with substantial presence in the cytosol, having different localization compared to endogenous DUSP11 (Fig 8). Despite this altered cellular distribution, vDUSP11 and host DUSP11 share a comparable ability to modulate the abundance of host RNAPIII transcripts. This further supports a model where endogenous RNAs can function to activate host PRRs.

In summary, we have identified a viral homolog of the host protein DUSP11 that has been co-opted by APVs/APV-related viruses. Future comparative studies with vDUSP11s may inform the role of host DUSP11 in shaping antiviral responses including functionally relevant amino acid residues. While the function during native APV/APV-related virus infection has yet to be elucidated, vDUSP11s share conserved functions in modulating the immune response, as seen with host DUSP11. This, along with the propensity for immune-related genes to be pirated, supports a role for both host and viral DUSP11s in modulating the innate immune response.

## Limitations of the study

The major limitation of this work is that the ability of vDUSP11s to reduce RIG-I signaling in a native virus genomic context such as avipox virus infection was not tested. Such experiments will be important for a complete understanding of vDUSP11. Consequently, it is incompletely understood whether vDUSP11s provide novel virus-beneficial activities or simply provide more of the same overall activity in addition to that provided by host DUSP11 during infection. Given that our cellular work was performed in human A549 cancer cells, an additional limitation of this study is that it remains unclear how applicable this model is to untransformed cells in the natural cellular context of avipoxvirus infection. To maximize sensitivity, our cellular work involves expressing vDUSP11 in cells lacking host DUSP11. It remains unknown how vDUSP11s will function in the presence of host DUSP11 during infection. We do demonstrate in the context of mutant poxvirus VACV *ΔE3L* infection that heterologous expression of vDUSP11s can modestly reduce RIG-I-mediated signaling. Nevertheless, it is currently unclear how the magnitude of these effects compares to during native avipox infection. It remains possible that in the context of native infection with appropriate timing and abundance of expression that the effects of vDUSP11 may be greater. Resolution of these issues awaits the development of a relevant avian model system for infection.

## Materials and methods

### Identification of avipoxvirus vDUSP11

Viral homologs to DUSP11 were identified using BLASTp [45] with the hDUSP11 sequence as query (S1 Table). Sequences were selected based on returned BLAST parameters (total score, e-value, and percent identity). Sequence alignment was generated using FASTA AA sequences acquired from NCBI database [41] and Clustal Omega [46] was used for multisequence alignment. In Fig 1B, sequence alignment was plotted using the R package ggmsa [47]. Reverse BLASTp [45] results for various vDUSP11 are shown in S2, S6, S7, and S9 Tables.

### Plasmids

The pcDNA3.1 puro and pLenti DUSP11-3xFLAG and DUSP11-3xFLAG-CM expression vectors were previously described [12]. The PISK-T7-HCV5’UTR vector was previously described [13]. vDUSP11 reference AA sequences were inputted into the IDT (Integrated DNA technologies) codon optimization tool to generate codon-optimized DNA sequences for expression in human cells. DNA sequences for SWPV2 vDUSP11, ChePV vDUSP11 and DUSP12 were synthesized as gBlocks (IDT) containing XhoI/XbaI restriction enzyme sites. The gBlocks were cloned into pcDNA3.1+ (puro) and plenti-EF1α (pLenti) backbones. Around-the-horn PCR [48] was utilized to add amino-terminal epitope (3xFLAG) tags to pLenti vDUSP11 and DUSP12 constructs. vDUSP11 catalytic mutant (CM) constructs were generated using overlap PCR [49] combined with restriction enzyme digest. Tagged pcDNA3.1+ (puro) 3xFLAG-tagged vDUSP11/vDUSP11-CM constructs were subcloned into pcDNA3.1 + behind a T7 promoter using restriction enzyme digest. For pGEX 4T-1 D11 CC AA: 29–205-GST, PCR was used to amplify a portion of hDUSP11 corresponding to amino acids 29–205 (Genbank accession #: NM_003584) using the pcDNA3.1-puro-3XFLAG-DUSP11 as template and this amplicon was cloned into pGEX-4T1 between the Not-I and BamHI restriction sites. For pGEX 4T-1 SWPV2 vD11 AA: 1–197-GST, PCR was used to amplify a portion of the vDUSP11 corresponding to amino acids 1–197 (Genbank accession #: ARE67303.1) using the plenti-EF1α SWPV2 vDUSP11 as template and cloned into pGEX-4T1 between the Not-I and BamHI restriction sites. For pGEX 4T-1 D11 CC-CM (C152S) AA: 29–205-GST, PCR was used to amplify a portion of hDUSP11 corresponding to amino acids 29–205 (Genbank accession #: NM_003584) using the pcDNA3.1-puro-3XFLAG-DUSP11-CM [12] as template and this amplicon was cloned into pGEX-4T1 between the Not-I and BamHI restriction sites. For pGEX 4T-1 SWPV2 vD11-CM (C135S) AA: 1–197-GST, PCR was used to amplify a portion of the viral DUSP11 corresponding to amino acids 1–197 (Genbank accession #: ARE67303.1) using the plenti-EF1α SWPV2-CM vDUSP11 as template and cloned into pGEX-4T1 between the Not-1 and BamHI restriction sites. Clones were confirmed using whole plasmid sequencing (Plasmidsaurus) or using Sanger sequencing.

### Cell lines

A549, BHK-21 and Vero cells were obtained from ATCC. A549 DUSP11 CRISPR–Cas9 targeted cell lines were previously described [12]. Cells were maintained in DMEM supplemented with 10% (v/v) fetal bovine serum (Corning) and 1% (v/v) penicillin-streptomycin (Corning). Generation of lentiviral particles, transduction and selection with blasticidin were performed as previously described [50]. A549 DUSP11 KO reconstituted cell lines were generated via transduction of lentiviral particles generated from the following lentiviral vectors: pLenti empty-vector (EV), pLenti DUSP11-3xFLAG, DUSP11 CM-3xFLAG, pLenti SWPV2 vDUSP11-3xFLAG, pLenti SWPV2 vDUSP11 CM-3xFLAG, pLenti ChePV vDUSP11-3xFLAG, pLenti ChePV vDUSP11 CM-3xFLAG, or pLenti DUSP12-3xFLAG as a negative control. For cells used in VACV infection experiments, A549 non-targeting (A549^NT^) [34], A549^∆RIG-I^ [34], A549^∆DUSP11+EV^, A549^∆DUSP11+DUSP11^, A549^∆DUSP11+SPXV DUSP11^, A549^∆DUSP11 +ChePV DUSP11^, and BHK-21 cells were cultured in Dulbecco’s modified eagle’s medium (DMEM) (Sigma, D6429) supplemented with 10% FBS (R&D, S12450), 1% antibiotics/antimycotics (Sigma, A5955), 1% MEM nonessential amino acids (Corning, 25–025-CI), and 1% L-glutamine (Corning, 25–005-CI).

### Virus strains and titration

Mutant VSV (rM51R-M-GFP) was provided by Douglas Lyles (Wake Forest School of Medicine, NC) and has been described previously [29]. Stocks were grown in BHK-21 cells and infectious virus concentration was determined using Vero cells. Wild-type vaccinia virus (VACV) (strain WR; obtained from Dr. Bernard Moss, NIH) and the WR VACV strain encoding a deletion of *E3L* (VACV *∆E3L*) [31] were propagated in BHK cells using low MOI infections. Virus stocks were titered on A549 cells using an immunofluorescence-based assay that detects VACV I3L protein staining as a readout for infection. Briefly, ~ 30,000 cells were seeded onto 35 mm glass coverslips and allowed to adhere overnight. Cells were then infected with serial dilutions of each virus stock for 8 hours, fixed with methanol, washed with PBS extensively, incubated at RT with blocking buffer (PBS with 1% BSA and 0.1% Triton X-100) for 1 hour, stained with mouse-anti-VV I3L antibody [51] for 2 hours, extensively washed with blocking buffer, incubated with Alexa Fluor 488-conjugated donkey anti-mouse secondary antibodies (Invitrogen Cat# A21202) for 1 hour, extensively washed with blocking buffer and then mounted onto glass slides using ProLong Diamond anti-fade with DAPI. Images were then captured on an Olympus Fv10i-LIV confocal microscope equipped with 405 nm and 463 nm lasers using a 60x objective.

### Phylogenetic analysis

DUSP11 AA sequences and related information were retrieved from Uniprot.org (ID: O75319). Accession numbers and related information for other atypical dual-specificity phosphatases for various host species were determined using HGNC [52] and sequences were determined using NCBI [41], Uniprot.org and BLASTp [45] for confirmation (S4 Table). AA sequences were retrieved from NCBI [41]. A multiple sequence alignment (MSA) of AA sequences were performed using Clustal Omega standard parameters implemented in Geneious prime with manual adjustments as needed (S9 Fig) [46]. Phylogenetic analysis was performed using PhyML with bootstrap replicates set to 100 [42]. Model selection was performed by Smart Model Selection (SMS) integrated into PhyML [53]. FigTree v1.4.4 (http://tree.bio.ed.ac.uk/software/figtree/) was used for visualization. Additional phylogenetic analysis was performed using the same parameters for host and avipox viral DUSP11 sequences (S8 Table). MSA of AA sequences was visualized using Jalview (S9 and S10 Figs) [54].

### Protein modeling

A partial crystal structure of hDUSP11 was obtained from wwPDB.org (PDB: 4JMJ) [25]. Alphafold2 was used to generate a full-length structure for hDUSP11, using standard parameters and the rank 1 structure was used for analysis. The crystal structure of hDUSP11 was superimposed on the Alphafold2 structural prediction for verification of solved regions, and the Alphafold2 structural prediction was ultimately used in depictions. Structural predictions of the SWPV2 vDUSP11 were generated using AlphaFold2 using standard parameters and the rank 1 structure was used for analysis [24]. UCSF chimera was used for visualization mapping, and analysis [55].

### In vitro transcription

To generate 5’-triphosphorylated HCV 5’ UTR RNA used for liposomal delivery and the *in vitro* phosphatase assays, *in vitro* transcription was performed with the AmpliScribe T7-Flash Transcription Kit (Bioresearch Technologies) according to manufacturer’s instructions. DNA templates for the *in vitro* transcription reaction were previously described [13]. RNA was then purified using MicroSpin G-25 columns [GE Healthcare].

### VSV infections and plaque assays

A549 DUSP11 KO cells reconstituted with various DUSP11/vDUSP11s were plated at a density of 0.1 x 10^6^ cells per well in a 12-well plate for virus infection. After 1 hour of virus adsorption with gentle shaking at 15-minute intervals, virus inoculum was aspirated from the wells and washed gently with PBS before adding growth medium. GFP images were taken at indicated time points, one representative image is shown with replicates shown in S11 Fig. 100 μL of media was collected at select time points post infection and stored at -80°C to determine the yield of infectious virus. Virus concentration was quantified by standard plaque assay on Vero cells in 6-well plate format, serially diluted in a countable range (5–250 plaques per well). 72 hours post-carboxymethyl cellulose (2.5%) overlay, plates were fixed and stained with 0.25% Coomassie blue in 10% acetic acid, 45% methanol or 0.5% methylene blue in 50% methanol.

### Vaccinia virus infections for analysis of ISG expression

A549 cells were either mock-infected or infected with either wild-type VACV (strain WR) or VACV*∆E3L* (MOI = 10) in serum-free DMEM for 1 hour. Viral inoculum was replaced after 1 hour with complete media. Cells were collected at 8 or 16 hpi for subsequent RNA extraction. Total RNA was extracted using the RNeasy Mini Kit (Qiagen, 74104) according to the manufacturer’s protocol. Subsequent RT-qPCR analysis was conducted on blinded samples.

### Northern blot analysis

Northern blot analysis was performed as previously described [56]. In brief, total RNA was harvested from cells using TRIzol reagent (Thermo Fisher) or PIG-B [56,57] and fractionated on 10–12% polyacrylamide gel electrophoresis (PAGE)-urea gel and transferred to Amersham Hybond-N+ membrane (GE Healthcare). Transferred membranes were crosslinked 2x with a UV crosslinker (UVP) with the RNA side of the membrane facing the UV source at 1,200 μJ/m^2^. Crosslinked membranes were prehybridized for ~ 1 hour at 55°C in hybridization solution (Takara). DNA oligos (S10 Table) were radiolabeled with [γ-^32^P] ATP (Perkin Elmer) by T4 polynucleotide kinase (New England Biolabs) for ~ 1 hour at 37°C. Radiolabeled probes were added to membranes following prehybridization and hybridized while rotating in UVP hybridization oven (HL-2000 HybriLinker UVP) overnight at 38°C. Probed membranes were exposed to a phosphor screen and visualized using Typhoon Biomolecular Imager (GE Healthcare). Membranes were stripped by incubating membrane with boiled 0.1% SDS with agitation for 15 minutes and repeated three times. Replicate northern blots shown in S12 Fig.

### GST protein purification

pGEX 4T-1 D11 CC AA: 29–205-GST, pGEX 4T-1 SWPV2 vD11 AA: 1–197-GST, pGEX 4T-1 D11 CC-CM (C152S) AA: 29–205-GST, pGEX 4T-1 SWPV2 vD11-CM (C135S) AA: 1–197-GST were expressed in NEB Express Iq *Escherichia coli* strain that produces large quantities of LacI repressor at 37°C. When OD600 reached 0.4, cells were induced with 0.05 mM isopropyl β-d-1-thiogalactopyranoside (IPTG) and subsequently grown for over 12 hours at 25°C. Cells were then incubated with lysis buffer (50 mM Hepes, pH 7.5, 500 mM NaCl, 10 mM imidazole, 5 mM β-mercaptoethanol, 0.1% (v/v) Triton X-100, and Roche Protease cOmplete EDTA Free Protease Inhibitor Cocktail. St.Louis, Missouri) and lysed by sonication (Q125 sonicator) on ice. Lysates were sonicated for 6 seconds three times with 1-minute breaks between each interval. The lysates were then centrifuged at 12,000 rcf for 45 minutes. The supernatant containing the GST fusion proteins were purified in batch by incubating lysates with 300 µ L glutathione sepharose 4B beads (Cytiva, Massachusetts) in PBS buffer for 4 hours at 4°C. The lysates were washed in tenfold volume of PBS for 8 rounds of washes. The washes were conducted at 4°C. Enzyme activity was initially assayed by *p*-nitrophenyl phosphate hydrolysis (Sigma-Aldrich. St.Louis, Missouri). The pNPP liquid was serially diluted (half dilutions) three times from the original 500mM stock concentration. 5 µ L of pNPP liquid was incubated with 5 µ L of purified protein preparations (GST, D11, SWPV, D11-CM, SWPV-CM) for 30 minutes at 37C in a 50 µ L reaction (50mM Tris pH 8.0, 50nM NaCl, 5mM DTT). 50 µ L of total reaction volume was added to each well of a 96 well plate was read by a microplate reader at an absorbance of 405 nm. The results showed an increase in absorbance for WT relative to the negative controls reactions (reaction buffer only, GST, D11-CM, SWPV-CM). Protein concentrations were estimated by comparing the intensity of the relevant Coomassie blue-stained band migrating at the appropriate size compared to known amounts of purified BSA protein. Proteins were visualized on a 12% SDS denaturing gel stained with coomassie blue. The gel was ran at 120V for 2 hours. The size migration ladder used is Color Protein Standard Broad Range (New England Biolabs, Massachusetts).

### In vitro phosphatase/XRN susceptibility assays

*In vitro* translated DUSP11, DUSP11-CM, SWPV2 vDUSP11, SWPV2 vDUSP11-CM lysates were generated using the TnT quick-coupled transcription/translation system (Promega) and the pcDNA3.1 DUSP11-3xFLAG, pcDNA3.1 DUSP11-CM-3xFLAG, pcDNA3.1 SWPV2-vDUSP11-3xFLAG, and pcDNA3.1 SWPV2-vDUSP11-CM-3xFLAG, plasmids as templates. Five micrograms of HCV 5’ UTR RNA was incubated in a 100-μL reaction [50mM Tris, 10mM KCl, 5mM DTT, 50mM EDTA, 40 units SUPERaseIn (Thermo Fisher Scientific)] with 6 μL of in vitro translated products (DUSP11-3xFLAG, DUSP11-CM-3xFLAG, SWPV2-vDUSP11-3xFLAG, SWPV2-vDUSP11-CM-3xFLAG, or a luciferase negative control) for 10 min at 37°C. EDTA was omitted from CIP control reactions as it is inhibitory of its enzymatic activity. RNA was purified using PIG-B [57]. Purified RNA from each phosphatase or control reaction was then used to set up two reactions 20 μL reactions containing 1 μg of treated RNA in NEBuffer 3.1 (New England Biolabs) with or without the addition of 1 μL (1U) recombinant XRN1 (New England Biolabs) and was then incubated for 60 minutes at 37°C as previously described [13]. The reactions were fractionated by 7.5% urea/PAGE and were then stained using ethidium bromide (~1 μg/mL) for 3–5 minutes while rocking. The Bio-Rad Molecular Imager GelDoc XR was then used to visualize gel via exposure to UV. Gel images were processed using Fiji (ImageJ variant) [58,59]. Replicate EtBr-stained urea gels shown in S13 Fig. For reactions where purified GST fusion proteins were assayed 10 µg of GST, 0.70 µg of GST-D11CC, 10 µg of GST-D11-CM, 0.50µg of GST-SWPV, or 2.25 µg of GST-SWPV-CM was added. In all experiments with purified proteins, increased amounts of CM variants and negative controls relative to WT proteins were utilized.

### 5pppRNA transfections

A549 DUSP11 KO reconstituted cells were seeded at a density of 0.1 X 10^6^ cells per well in a 12-well format. 24 hours following plating, cells were transfected with 5–10 ng of *in vitro* transcribed 5’-triphosphorylated HCV 5’ UTR RNA, or with Lipofectamine 2000 (Invitrogen) alone control (3 µ L). Transfection was terminated after 18 hours by the addition of either TRIzol reagent (Invitrogen) or PIG-B, followed by RNA extraction and subsequent RT-qPCR analysis.

### Real-time PCR (RT-qPCR)

Total RNA was extracted from cells using TRIzol reagent or PIG-B [57]. Extracted RNA was treated with DNase I (Thermo Fisher) followed by ethanol precipitation (100% ethanol, 3M sodium acetate). Precipitated RNA was resuspended in nuclease-free water (Thermo Fisher) and was quantified using the NanoDrop ND-1000 Spectrophotometer. RNA purity was assessed using the measured OD at 260/280 nm, with RNA considered acceptable having values within ~1.7-2.1. Complementary DNA (cDNA) was synthesized using SuperScript III reverse transcriptase (Invitrogen) using equal amounts of RNA. All RT-qPCR quantification and measurements were performed on a StepOnePlus Real-Time PCR system (Applied BioSystems). A standard 3-step PCR cycling protocol (95°C for 15 sec, 60°C for 15 sec, 68°C for 30 sec, 40 cycles) using PerfeCTa SYBR Green FastMix (Quantabio) was used according to the manufacturer’s instructions. At least two technical replicates per sample/condition. Gene expression of human IFNB1 and ISG15 were normalized to the expression of GAPDH (S10 Table). GAPDH values for A549 DUSP11 KO + EV cells transfected with L2K alone were used when defining the experiment negative control within the Applied BioSystems program [14].

### Confocal images

A549 DUSP11 KO reconstituted lines were plated at 0.5 X 10^5^ cells per well in a 24-well plate containing a 12-mm glass coverslip. The next day cells were washed three times with 1X PBS before fixation with 2% paraformaldehyde for 20 minutes. Paraformaldehyde was removed and cells were washed three times with 1X PBS. Following washes, cells were permeabilized with 0.5% Triton X-100 in 1X PBS 3% BSA for 2 minutes at room temperature. Next, the permeabilization buffer was removed and cells were incubated in blocking buffer (0.2% Triton X-100, 3% BSA, 1X PBS) while rocking for 1 hour at room temperature. Following incubation, M2 FLAG antibody (Sigma Aldrich #F1804) was diluted to 1:1000 in blocking buffer and incubated overnight at 4°C. The next day cells were subjected to three 5-minute washes in blocking buffer. Secondary antibodies (S10 Table) were diluted 1:1000 in blocking buffer and added to cells to incubate for 30 minutes at room temperature in the dark. Cells were washed three times in blocking buffer for 5 minutes each. ProLong Diamond Antifade Mountant (Invitrogen #P36961) was used when mounting coverslips on slides. Cells were imaged on ZEISS confocal using the 40x objective (1 representative image shown). Images were processed to final form using ZEISS image analysis software and Image J.

### Immunoblot analysis

Protein was extracted from cells using either RIPA lysis buffer (50 mM Tris-HCl, 150 mM NaCl, 0.25% sodium-deoxycholate, 1% NP-40, 0.1% SDS with protease inhibitor) supplemented with Protease and Phosphatase Inhibitor Cocktail (Abcam, ab201119) or SDS page sample buffer (1X) [60]. Cell lysate was fractionated on an 8% SDS-PAGE and transferred to Amersham Protran 0.45 μm nitrocellulose membrane (GE Healthcare). Blots were incubated in blocking buffer (Intercept PBS blocking buffer, LI-COR) then probed with primary antibodies for M2 FLAG (monoclonal, 1:2000 dilution, Sigma Aldrich, F1804) and beta-Tubulin (polyclonal, 1:2000 dilution, Cell Signaling, 2146S) in blocking buffer overnight at 4°C. Blots were washed with ~10 mL 1X PBST three times for 5 minutes prior to incubation with secondary antibodies. IRDye 800CW (1:10,000 dilution, LI-COR, 926–32213) and IRDye 680LT (1:10,000 dilution, LI-COR, 926–68022) were used as secondary antibodies, diluted in blocking buffer. Blots were washed with ~ 10mL 1X PBST three times for 5 minutes, then imaged with an Odyssey CLx infrared imaging system (LI-COR). Uncropped western blots shown in S14 Fig.

### Synteny analysis

NCBI was used to identify genomic locations for viral DUSP11 sequences within the viral genomes [41]. Accession numbers for adjacent coding portions corresponding to the 5 genes upstream and downstream from each viral DUSP11 were recorded (S5 Table). Reciprocal BLAST hits (RBH) were performed for coding sequences of vDUSP11-adjacent genes across virus species to identify homologs (S5 Table) [45]. Genomic database annotations and the NCBI database were used for gene identities and homology [41]. CoreGenes 5.0 was used to further verify the presence of homologous genes between selected poxvirus genomes [61].

### Statistical analysis

GraphPad Prism software was used for statistical analyses. For Fig 5, a standard unpaired student t-test was used on transformed (Y = log(X)) viral titers. For Fig 6, panels A and B were analyzed using a standard unpaired students t-test, panels C and D were analyzed using one-way ANOVA followed by Dunnett’s multiple comparison test. Results for Dunnett’s multiple comparison test are indicated on corresponding figures. ANOVA results for VACV infected samples for panels C and D were *P* = 0.01 and *P* = 0.08, respectively. For S6 Fig, panels C and D were analyzed using a standard unpaired students t-test, panels G and H were analyzed using one-way ANOVA followed by Dunnett’s multiple comparison test. Results for Dunnett’s multiple comparison test are indicated on corresponding figures. ANOVA results for panels G and H were *P* = 0.07 and *P* = 0.16, respectively. For all other statistical analysis, a standard Student *t*-test was used with significance set at *P* = 0.05. Error bars were presented as mean ± standard error of the mean. Statistical consultation was provided by the Department of Statistics and Data Sciences at the University of Texas at Austin.

## Supporting information

S1 Fig Predicted local distance difference test (PLDDT) results for generated Alphafold2 structures in Fig 1. pLDDT for Alphafold2 [24] structural prediction for (A) human DUSP11 (hDUSP11) and (B) SWPV2 vDUSP11 (SWPV2) per amino acid position, shown on x-axis. Rank 1 structure for both sequences was selected for visualization in Fig 1. Higher pIDDT values (y-axis) indicate higher confidence levels for structural predictions.
(TIF)

S2 Fig A broad phylogenetic distribution of avipox and avipox-related poxviruses containing vDUSP11. (A) A graphical representation of *hDUSP11* with AA sequence length. (B) A cladogram built from published phylogenetic data [63], focusing on a subset of poxviruses encoding vDUSP11. Sub-clades (B1 and B3) are designated according to Gyuranecz et al. (2013) [64]. Graphical representations of *vDUSP11* are to the right of each virus, demonstrating variations in vDUSP11 AA sequence length between viruses. Magpiepox virus 2 and crowpox virus encode truncated vDUSP11 as indicated. The presence of * indicates unclassified poxviruses. vDUSP11 p-loop indicated in green.
(TIF)

S3 Fig Sequence alignment of vDUSP11 sequences with consensus identity. (TIF)

S4 Fig Purified vDUSP11 sensitizes HCV 5’ UTR RNA to XRN-mediated degradation. GST fusion hDUSP11 catalytic-core (D11/D11-CC) (AA: 29–205), SWPV2 vDUSP11 (SWPV) (AA: 1–197) and their corresponding catalytic mutants (D11-CM and SWPV-CM, respectively) confirm vDUSP11 catalytic activity of purified proteins as demonstrated in Fig 3 for full length proteins in *in vitro* translated lysates. (A). Protein preparations were separated on an SDS PAGE gel and stained with Coomassie blue. Image shows the GST-vDUSP11 preparation is approximately >95% pure. Reactions in panel B and C purposely contain excess amounts of negative control GST and catalytically inactive (CM) variants. For comparison, different amounts of purified BSA protein are shown on the right side of the gel. (B) Representative gel of XRN sensitivity assay. RNA corresponding to the 5’ end of the HCV genome was incubated with GST or various GST- fusion proteins, RNA was purified and then exposed to XRN1. Resultant product RNAs were ran on a gel and stained with ethidium bromide. The same amount of proteins loaded in panel A are used in the reactions. A size marker ladder is shown on the left side of the gel. (C) Graphical representation of the ratio of the XRN1-cleaved:full length HCV RNA fragment following treatment with recombinant XRN1 (+XRN1/-XRN1) as an indirect measure of the proportion that is converted to the monophosphate form. Results are represented relative to the reaction buffer alone (Rxn Buffer). Data are derived from n = 5 independent replicates. In all panels, data are represented as mean ± SEM.
(TIF)

S5 Fig vDUSP11 modulates immune activation in response to liposomal 5’-ppp-RNAs Confirmation of key result trends from Fig 4 via a different wet bench scientist. A549 DUSP11 knockout (KO) reconstituted cells (12-well) were transfected with 5–10 ng of *in vitro* transcribed 5’-ppp-RNA for 18 hours followed by RT-qPCR to assay induction of ISGs. (A) RT-qPCR analysis of *ISG15* and (B) *IFNB1* mRNA normalized to *GAPDH* mRNA. Results are represented relative to those of empty vector-expressing cells. Data are derived n = 1 replicates.
(TIF)

S6 Fig Absence of either RIG-I or DUSP11 results in subtle differences in VACV replication. Viral titers from cells infected with either wild-type recombinant vaccinia virus (VACV) (WT-R), or an ∆ *E3L* deficient VACV (∆*E3L*) at an MOI of 10. At 16 hours post infection (hpi) viral supernatant was collected and titer was quantified by detection of the VACV I3L protein (VV-I3L) via immunofluorescence. Previously characterized A549 non-targeted (NT) and A549 RIG-I knock out cells (∆RIG-I) [34] were infected by either (A) WT-R or (B) ∆*E3L* VACV. Results from (A) and (B) are represented relative to input levels in (C) and (D), respectively. Viral titers from A549 DUSP11 knock out cells (∆DUSP11) stability expressing either an empty vector plasmid (EV), human DUSP11 (DUSP11), SWPV2 vDUSP11 (SWPV), or ChePV vDUSP11 (ChePV) after infection by either (E) WT-R or (F) ∆*E3L* VACV. Results from (E) and (F) are represented relative to input levels in (G) and (H), respectively. For all panels, data are derived from n = 3 independent replicates. All data are represented as mean ± SEM. (*) *P *< 0.05. For panels C and D, two-tailed Student’s *t*-tests were used for analysis. For panels G and H, one-way ANOVAs were performed, followed by Dunnett’s multiple comparison test with EV set as the control for comparison. ANOVAs failed to rise above statistical significance, *P*-values from the Dunnett’s test are represented in the figure.
(TIF)

S7 Fig vDUSP11 modulates steady-state RNA levels of endogenous RNAP III transcripts. Confirmation of key result trends from Fig 7 via a different wet bench scientist. (A) Northern blot analysis of vtRNA1–1 and vtRNA2–1 using RNA from A549 DUSP11 KO reconstituted cells. (B) Graphical representation of relative band intensity of vtRNA1–1 and vtRNA2–1 normalized to the relative band intensity of the cysteine-tRNA. Values are represented relative to the A549 DUSP11 KO + EV cell line. Data are derived from n = 1 replicates.
(TIF)

S8 Fig An inferred phylogenetic tree highlighting likely avian origin of APV/AdjPV vDUSP11s. An inferred tree built using 26 host and viral DUSP11 amino acid (AA) sequences by maximum-likelihood analysis phylogenetic tree using PhyML [42]. AA sequences for host DUSP11s and APV/AdjPV putative vDUSP11s were aligned using Clustal Omega (S10 Fig) [46]. Clustal alignment was used to run PhyML analysis with the Q.plant +G + I model selected by SMS [53]. Sequences were retrieved from the NCBI sequence database [41] and Uniprot (www.uniprot.org/) (S8 Table). Putative APV/AdjPV vDUSP11s (orange) cluster with avian host DUSP11s. 100 bootstrap replicates were performed; branch support ≥50% (*) or ≥ 70% (**) are indicated. The lamprey DUSP11 was specified as the outgroup.
(TIF)

S9 Fig Clustal Omega alignment of host/poxviral DUSP sequences (trimmed) used for PhyML analysis for Fig 2. Position of catalytic cysteine indicated by red arrow. Position of catalytic cysteine indicated by red arrow.
(TIF)

S10 Fig Clustal Omega alignment of host DUSP11 and avipox vDUSP11 sequences (trimmed) used for PhyML analysis for S8 Fig. Position of catalytic cysteine indicated by red arrow. (TIF)

S11 Fig M51R VSV infection GFP images. (TIF)

S12 Fig Uncropped northern blots. (TIF)

S13 Fig Uncropped ethidium bromide-stained urea gels. (TIF)

S14 Fig Uncropped western blots. (TIF)

S1 Table BLASTp results using human DUSP11 (uniprot o75319) as query limiting results to viruses (taxid: 10239). (XLSX)

S2 Table BLASTp results using vDUSP11 from SWPV2 as query (ARE67303.1). (XLSX)

S3 Table Avipox and avipox-related viral DUSP11 accession IDs. *trucated portion with homology to other vDUSP11. **identified through blast of genome sequence for CNPV vDUSP11. No annotated ORF, however complete coding region present including start and stop codon. 73.9% similarity to CNPV vDUSP11.
(XLSX)

S4 Table Accession IDs used for phylogenetic analysis of related host and poxviral DUSPs for Fig 2. (XLSX)

S5 Table Genes surrounding the genomic locations of avipox vDUSP11 from synteny analysis. *Description refers to putative ORF identity determined through homology analysis or as directly indicated by NCBI [**39**] entry.
(XLSX)

S6 Table BLASTp results using penguinpox virus 2 (PEPV2) viral DUSP11 as query (QRM15716.1). (XLSX)

S7 Table BLASTp results using vDUSP11 for mudlarkpox virus as query (QRM15361.1). (XLSX)

S8 Table Accession IDs used to retrieve FASTA sequence for construction of DUSP11 phylogenetic tree in S8 Fig. (XLSX)

S9 Table Reverse BLASTp results using remaining vDUSP11 as query. vDUSP11 used as query is listed first in each table. First 10 results shown for each virus excluding other avipox vDUSP11s.
(XLSX)

S10 Table Sequence information, synthetic oligonucleotides and DNA, and reagents. (XLSX)
