# Supplementary figures and images for: Viral piracy of host RNA phosphatase DUSP11 by avipoxviruses
Source: PLoS Pathog. 2025 Apr 21;21(4):e1013101. doi: 10.1371/journal.ppat.1013101 (PMC12058148; doi:10.1371/journal.ppat.1013101)

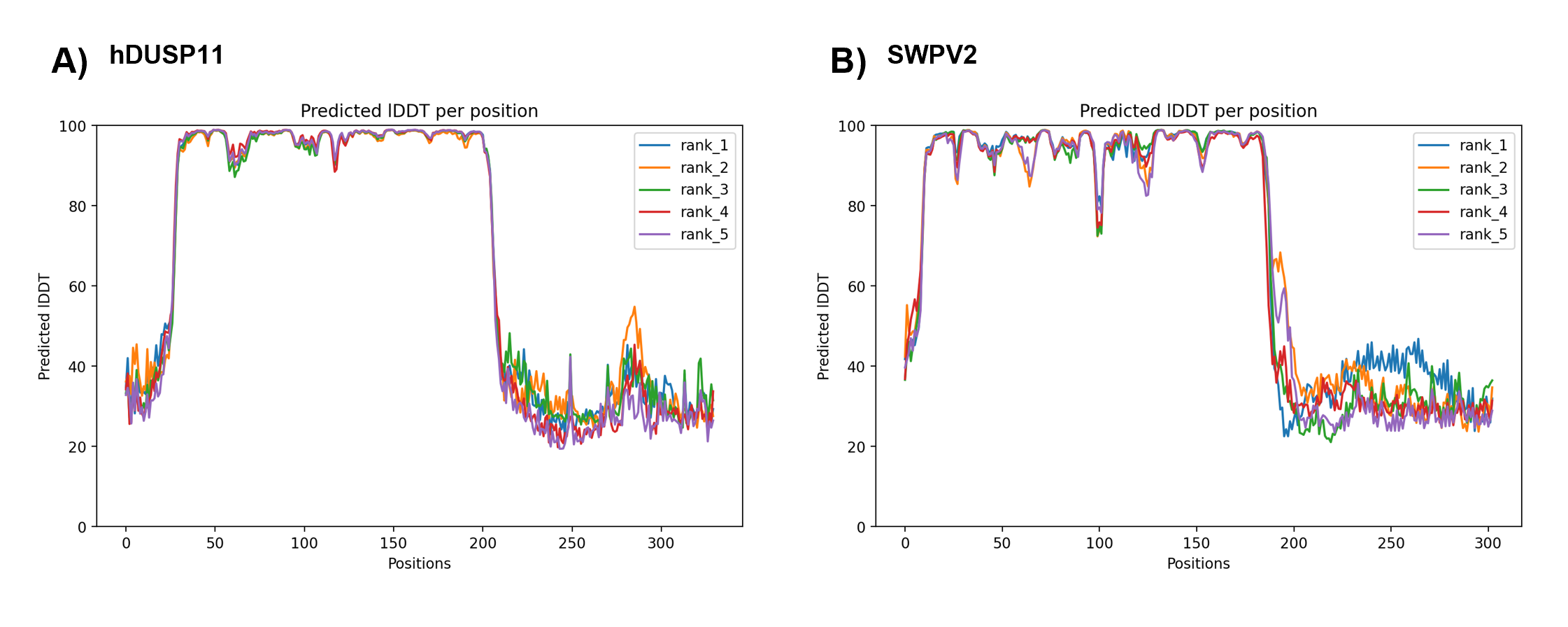

Supplement: S1 Fig — Rank 1 structure for both sequences was selected for visualization in Fig 1. Higher pIDDT values (y-axis) indicate higher confidence levels for structural predictions. (TIF) [file ppat.1013101.s001.tif]

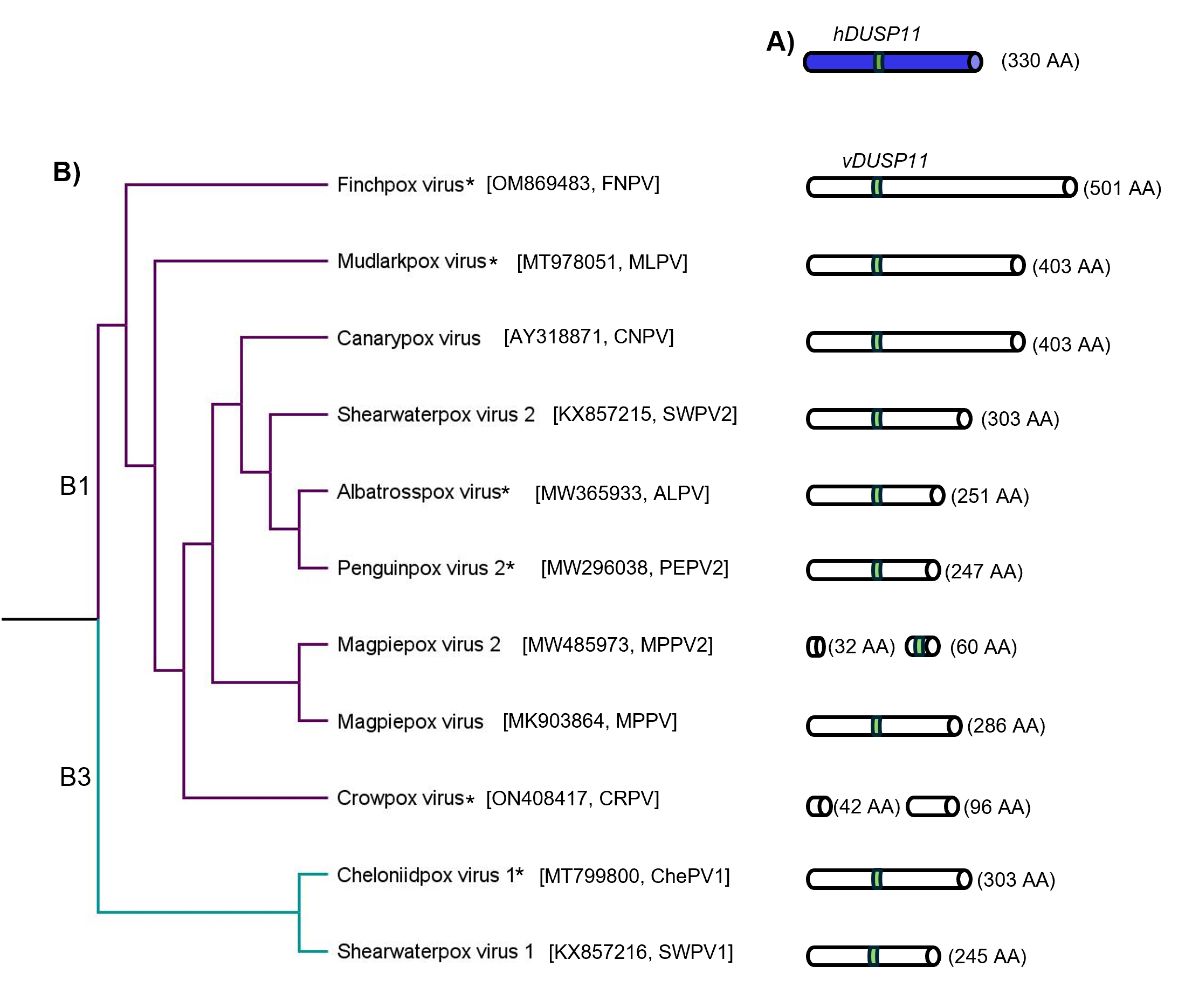

Supplement: S2 Fig — (A) A graphical representation of hDUSP11 with AA sequence length. (B) A cladogram built from published phylogenetic data [63], focusing on a subset of poxviruses encoding vDUSP11. Sub-clades (B1 and B3) are designated according to Gyuranecz et al. (2013) [64]. Graphical representations of vDUSP11 are to the right of each virus, demonstrating variations in vDUSP11 AA sequence length between viruses. Magpiepox virus 2 and crowpox virus encode truncated vDUSP11 as indicated. The presence of * indicates unclassified poxviruses. vDUSP11 p-loop indicated in green. (TIF) [file ppat.1013101.s002.tif]

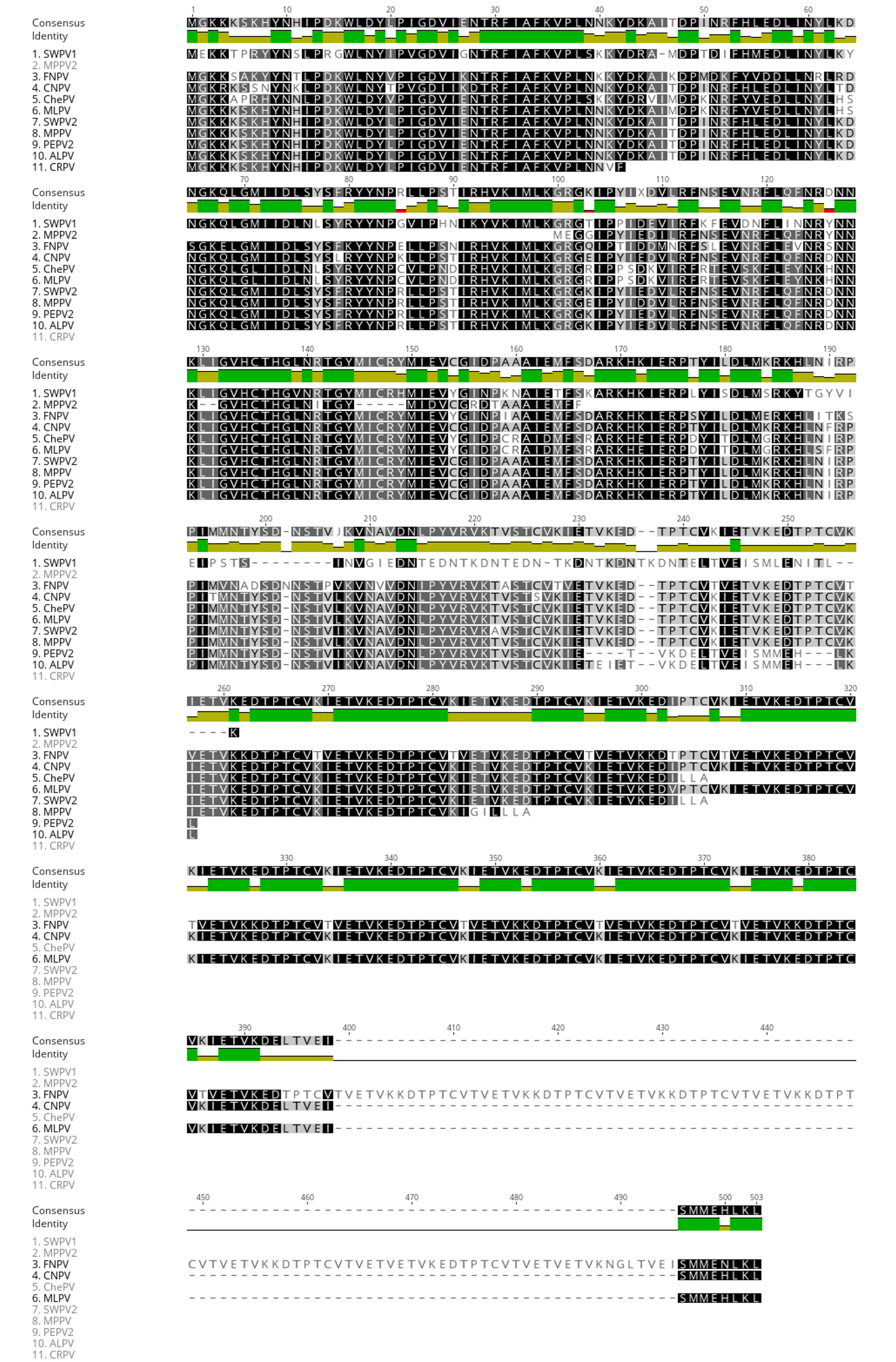

Supplement: S3 Fig — (TIF) [file ppat.1013101.s003.tif]

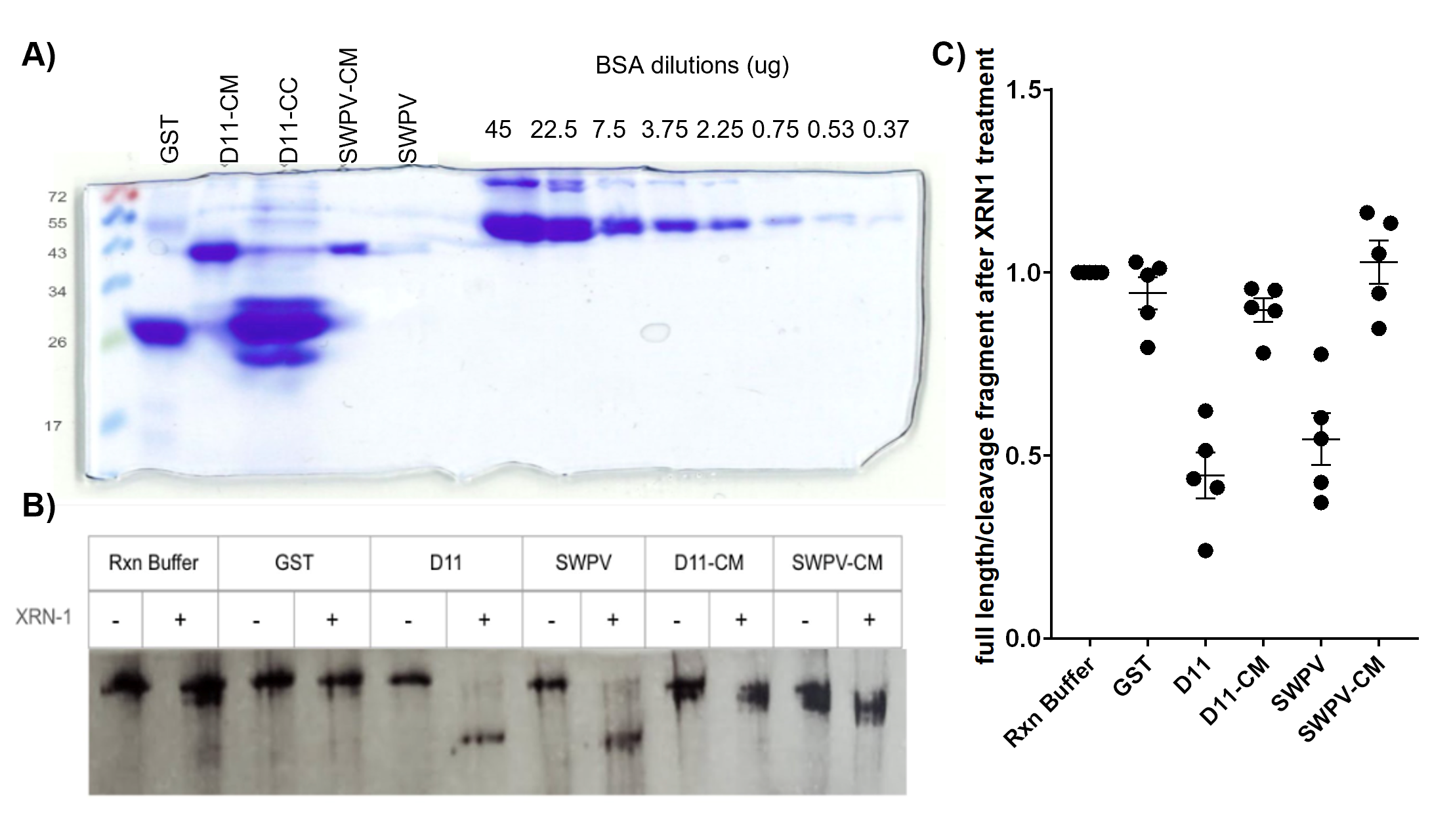

Supplement: S4 Fig — GST fusion hDUSP11 catalytic-core (D11/D11-CC) (AA: 29–205), SWPV2 vDUSP11 (SWPV) (AA: 1–197) and their corresponding catalytic mutants (D11-CM and SWPV-CM, respectively) confirm vDUSP11 catalytic activity of purified proteins as demonstrated in Fig 3 for full length proteins in in vitro translated lysates. (A). Protein preparations were separated on an SDS PAGE gel and stained with Coomassie blue. Image shows the GST-vDUSP11 preparation is approximately >95% pure. Reactions in panel B and C purposely contain excess amounts of negative control GST and catalytically inactive (CM) variants. For comparison, different amounts of purified BSA protein are shown on the right side of the gel. (B) Representative gel of XRN sensitivity assay. RNA corresponding to the 5’ end of the HCV genome was incubated with GST or various GST- fusion proteins, RNA was purified and then exposed to XRN1. Resultant product RNAs were ran on a gel and stained with ethidium bromide. The same amount of proteins loaded in panel A are used in the reactions. A size marker ladder is shown on the left side of the gel. (C) Graphical representation of the ratio of the XRN1-cleaved:full length HCV RNA fragment following treatment with recombinant XRN1 (+XRN1/-XRN1) as an indirect measure of the proportion that is converted to the monophosphate form. Results are represented relative to the reaction buffer alone (Rxn Buffer). Data are derived from n = 5 independent replicates. In all panels, data are represented as mean ± SEM. (TIF) [file ppat.1013101.s004.tif]

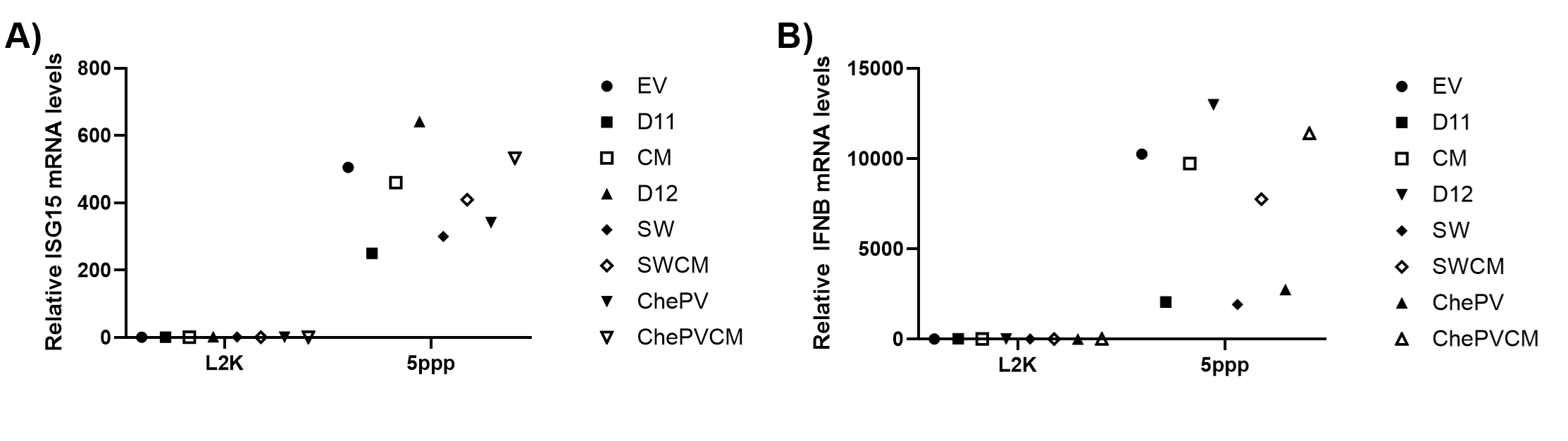

Supplement: S5 Fig — Confirmation of key result trends from Fig 4 via a different wet bench scientist. A549 DUSP11 knockout (KO) reconstituted cells (12-well) were transfected with 5–10 ng of in vitro transcribed 5’-ppp-RNA for 18 hours followed by RT-qPCR to assay induction of ISGs. (A) RT-qPCR analysis of ISG15 and (B) IFNB1 mRNA normalized to GAPDH mRNA. Results are represented relative to those of empty vector-expressing cells. Data are derived n = 1 replicates. (TIF) [file ppat.1013101.s005.tif]

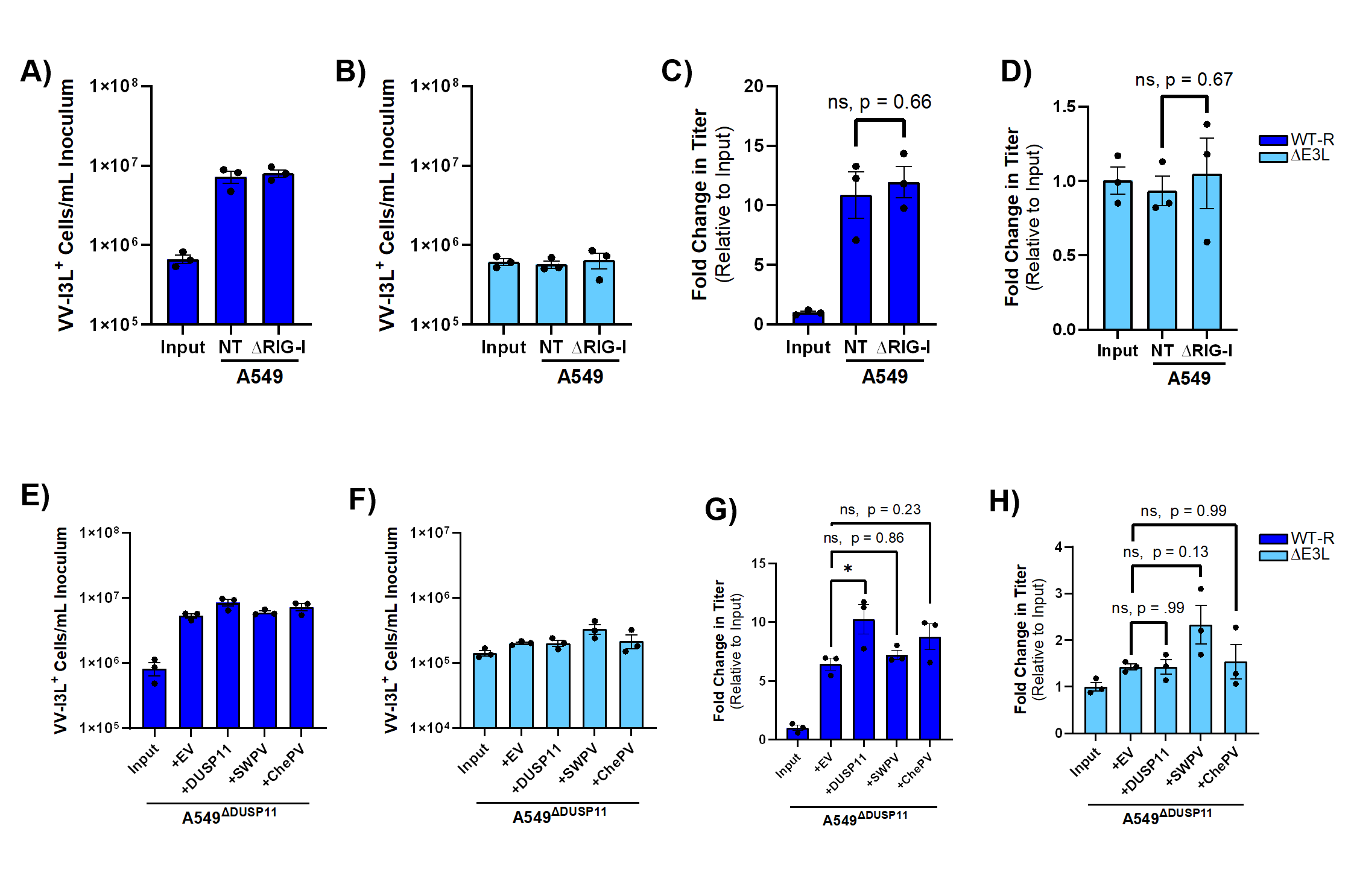

Supplement: S6 Fig — Viral titers from cells infected with either wild-type recombinant vaccinia virus (VACV) (WT-R), or an ∆ E3L deficient VACV (∆E3L) at an MOI of 10. At 16 hours post infection (hpi) viral supernatant was collected and titer was quantified by detection of the VACV I3L protein (VV-I3L) via immunofluorescence. Previously characterized A549 non-targeted (NT) and A549 RIG-I knock out cells (∆RIG-I) [34] were infected by either (A) WT-R or (B) ∆E3L VACV. Results from (A) and (B) are represented relative to input levels in (C) and (D), respectively. Viral titers from A549 DUSP11 knock out cells (∆DUSP11) stability expressing either an empty vector plasmid (EV), human DUSP11 (DUSP11), SWPV2 vDUSP11 (SWPV), or ChePV vDUSP11 (ChePV) after infection by either (E) WT-R or (F) ∆E3L VACV. Results from (E) and (F) are represented relative to input levels in (G) and (H), respectively. For all panels, data are derived from n = 3 independent replicates. All data are represented as mean ± SEM. (*) P < 0.05. For panels C and D, two-tailed Student’s t-tests were used for analysis. For panels G and H, one-way ANOVAs were performed, followed by Dunnett’s multiple comparison test with EV set as the control for comparison. ANOVAs failed to rise above statistical significance, P-values from the Dunnett’s test are represented in the figure. (TIF) [file ppat.1013101.s006.tif]

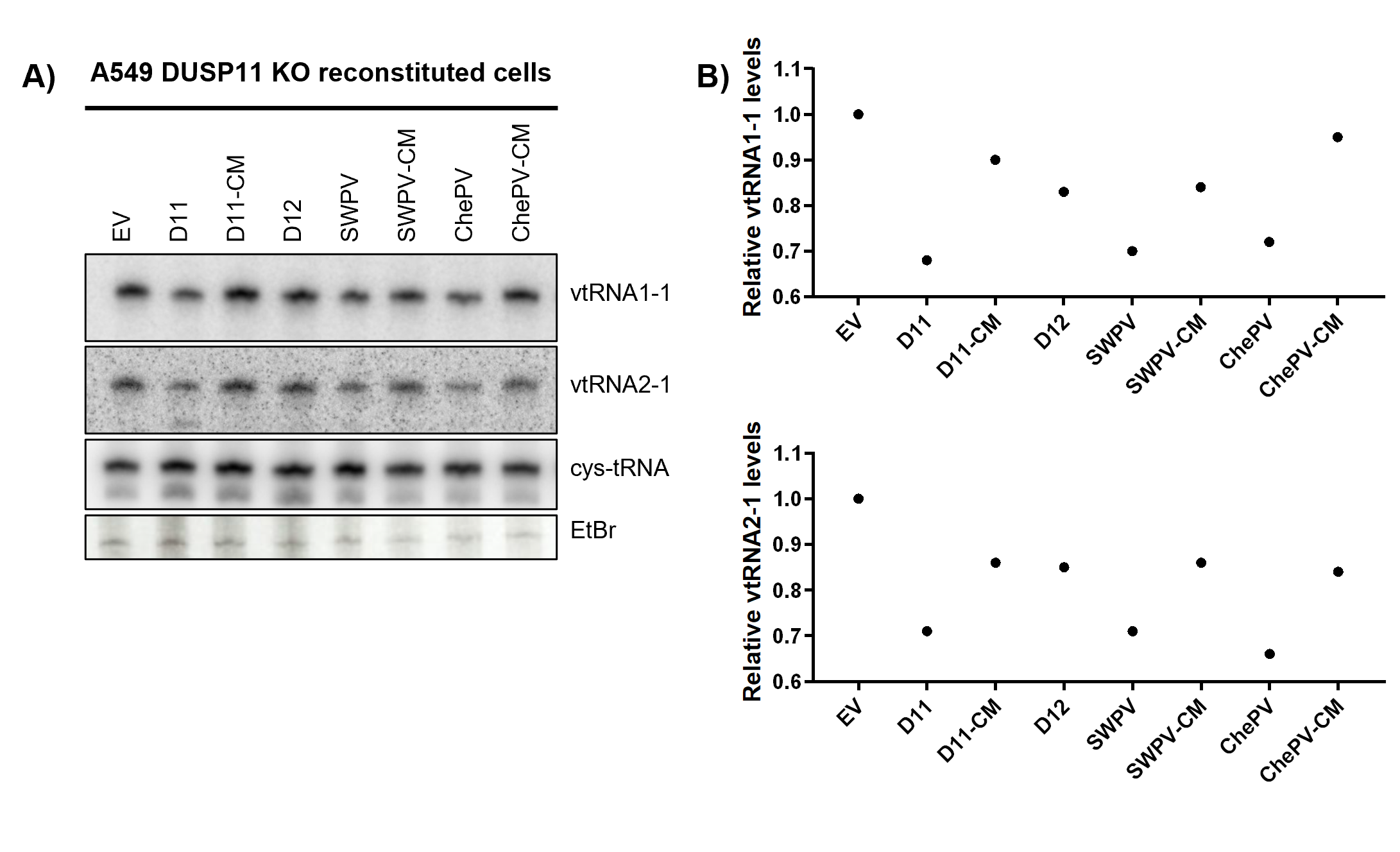

Supplement: S7 Fig — Confirmation of key result trends from Fig 7 via a different wet bench scientist. (A) Northern blot analysis of vtRNA1–1 and vtRNA2–1 using RNA from A549 DUSP11 KO reconstituted cells. (B) Graphical representation of relative band intensity of vtRNA1–1 and vtRNA2–1 normalized to the relative band intensity of the cysteine-tRNA. Values are represented relative to the A549 DUSP11 KO + EV cell line. Data are derived from n = 1 replicates. (TIF) [file ppat.1013101.s007.tif]

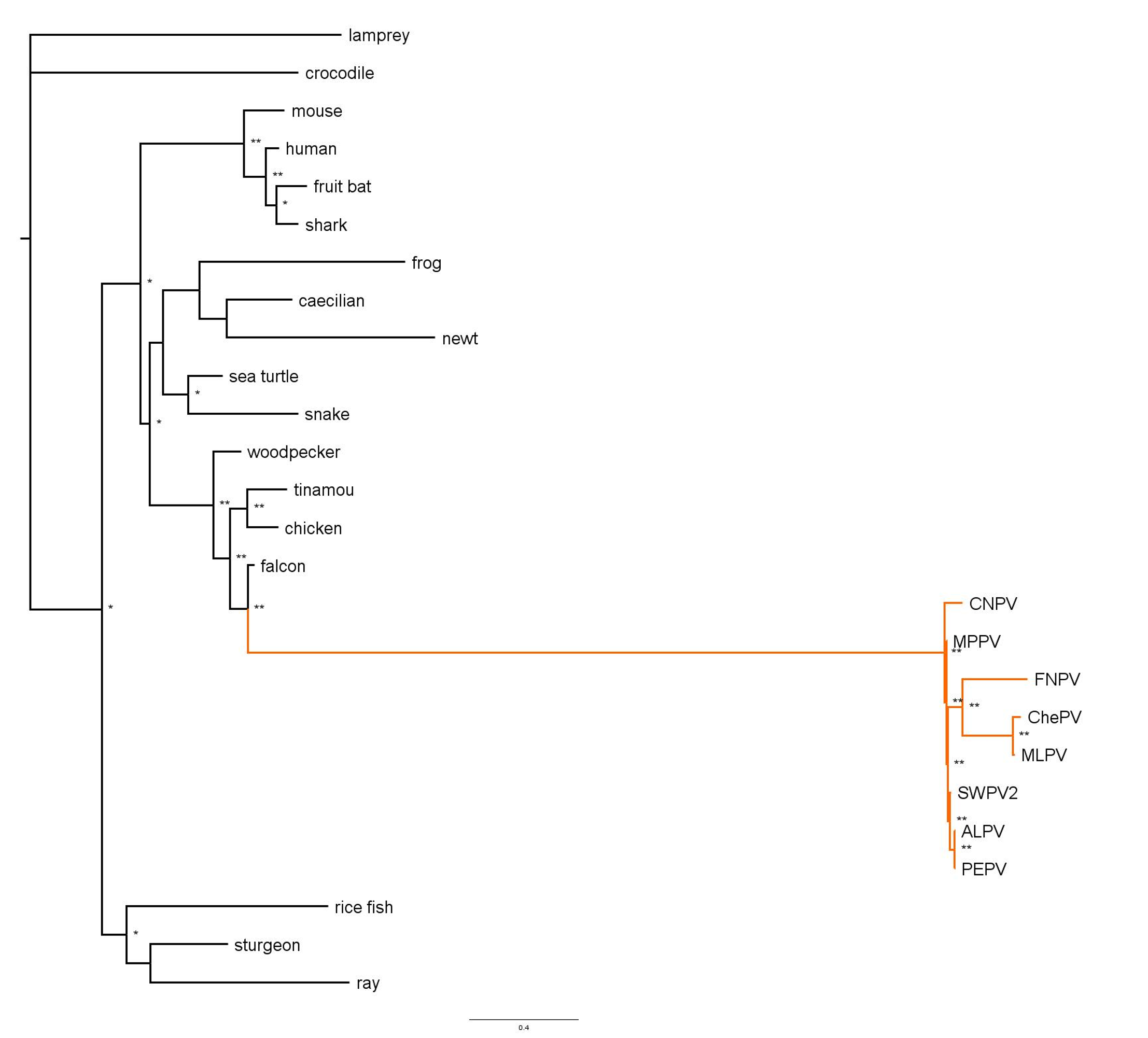

Supplement: S8 Fig — An inferred tree built using 26 host and viral DUSP11 amino acid (AA) sequences by maximum-likelihood analysis phylogenetic tree using PhyML [42]. AA sequences for host DUSP11s and APV/AdjPV putative vDUSP11s were aligned using Clustal Omega (S10 Fig) [46]. Clustal alignment was used to run PhyML analysis with the Q.plant +G + I model selected by SMS [53]. Sequences were retrieved from the NCBI sequence database [41] and Uniprot (www.uniprot.org/) (S8 Table). Putative APV/AdjPV vDUSP11s (orange) cluster with avian host DUSP11s. 100 bootstrap replicates were performed; branch support ≥50% (*) or ≥ 70% (**) are indicated. The lamprey DUSP11 was specified as the outgroup. (TIF) [file ppat.1013101.s008.tif]

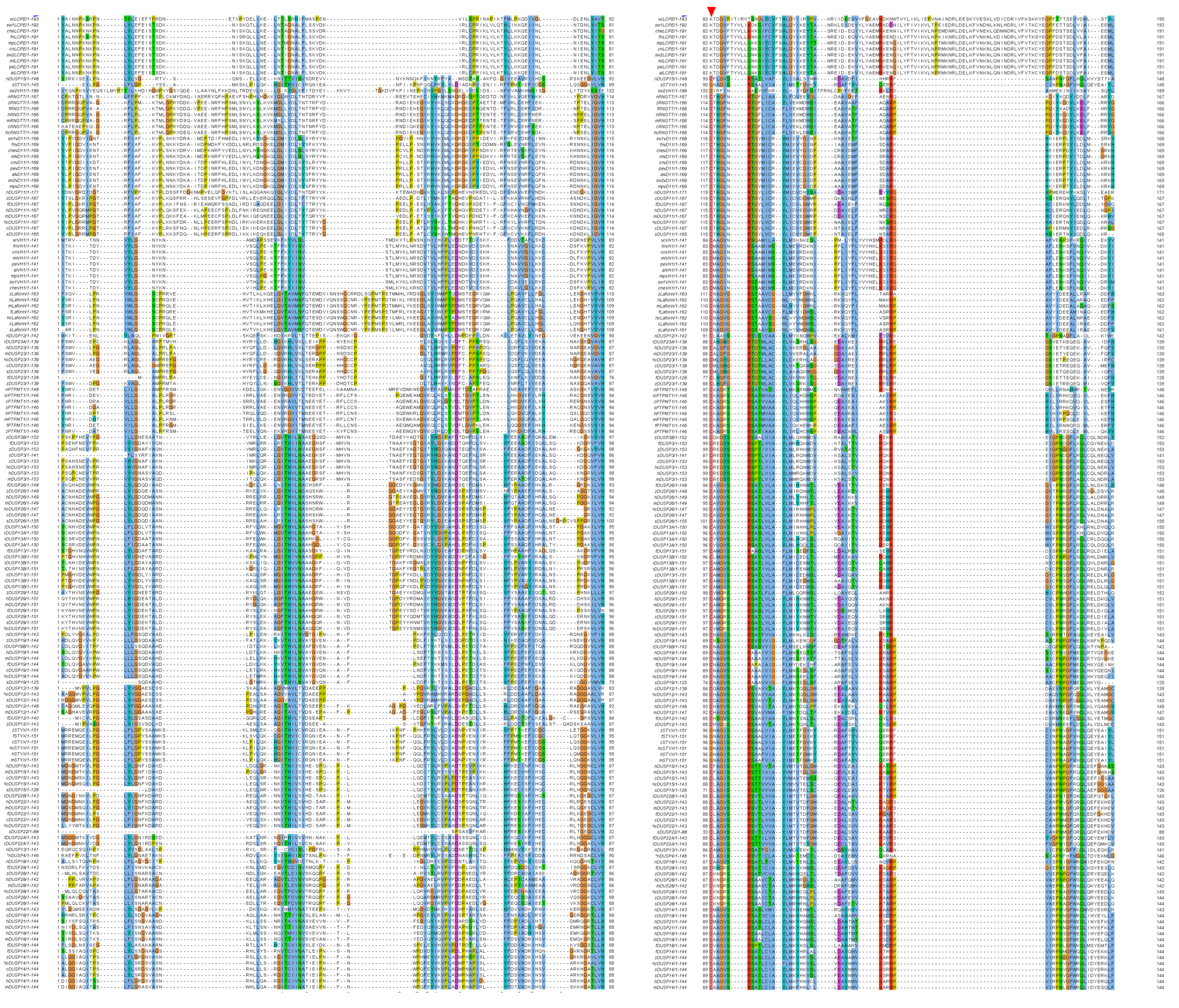

Supplement: S9 Fig — Position of catalytic cysteine indicated by red arrow. (TIF) [file ppat.1013101.s009.tif]

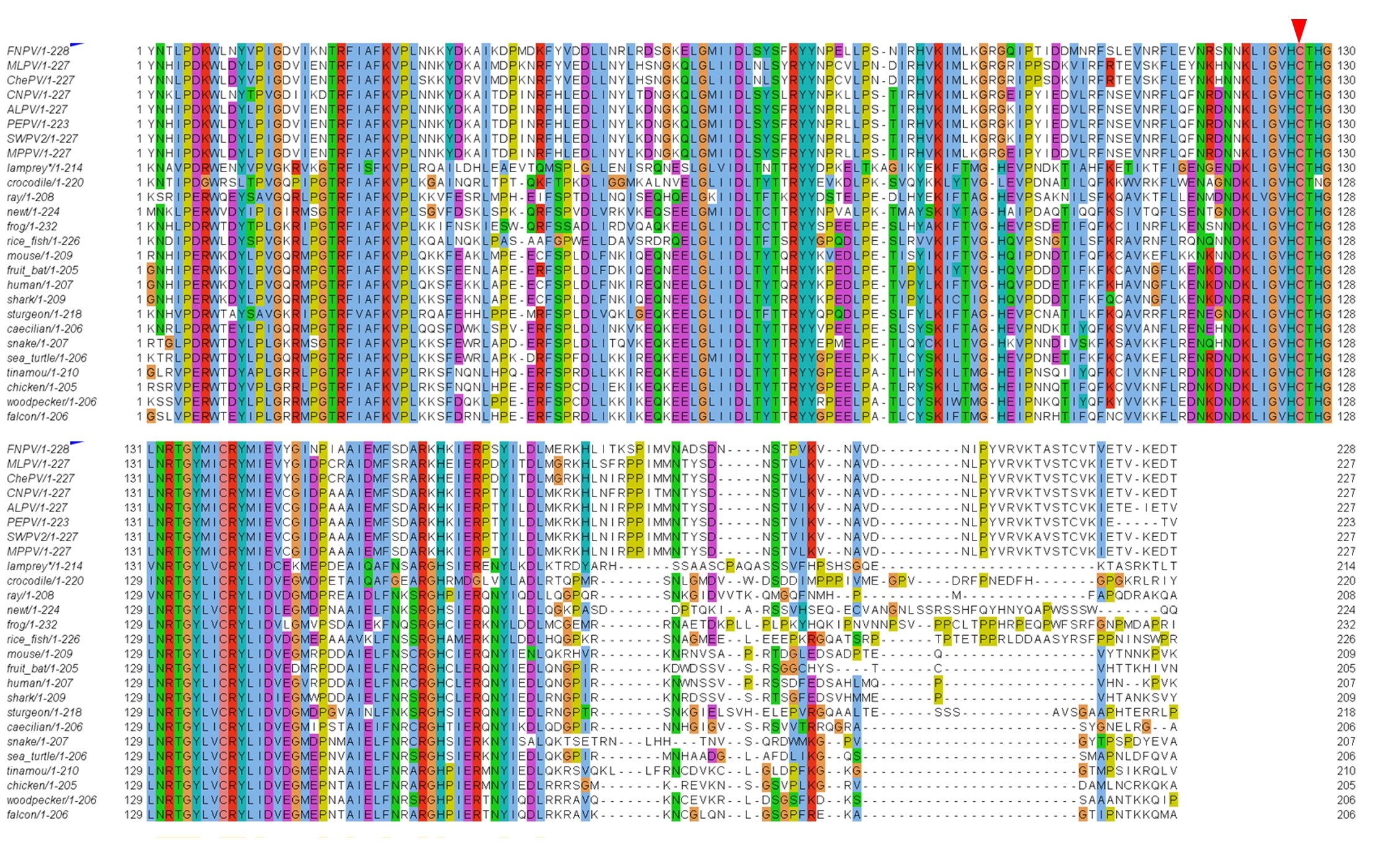

Supplement: S10 Fig — (TIF) [file ppat.1013101.s010.tif]

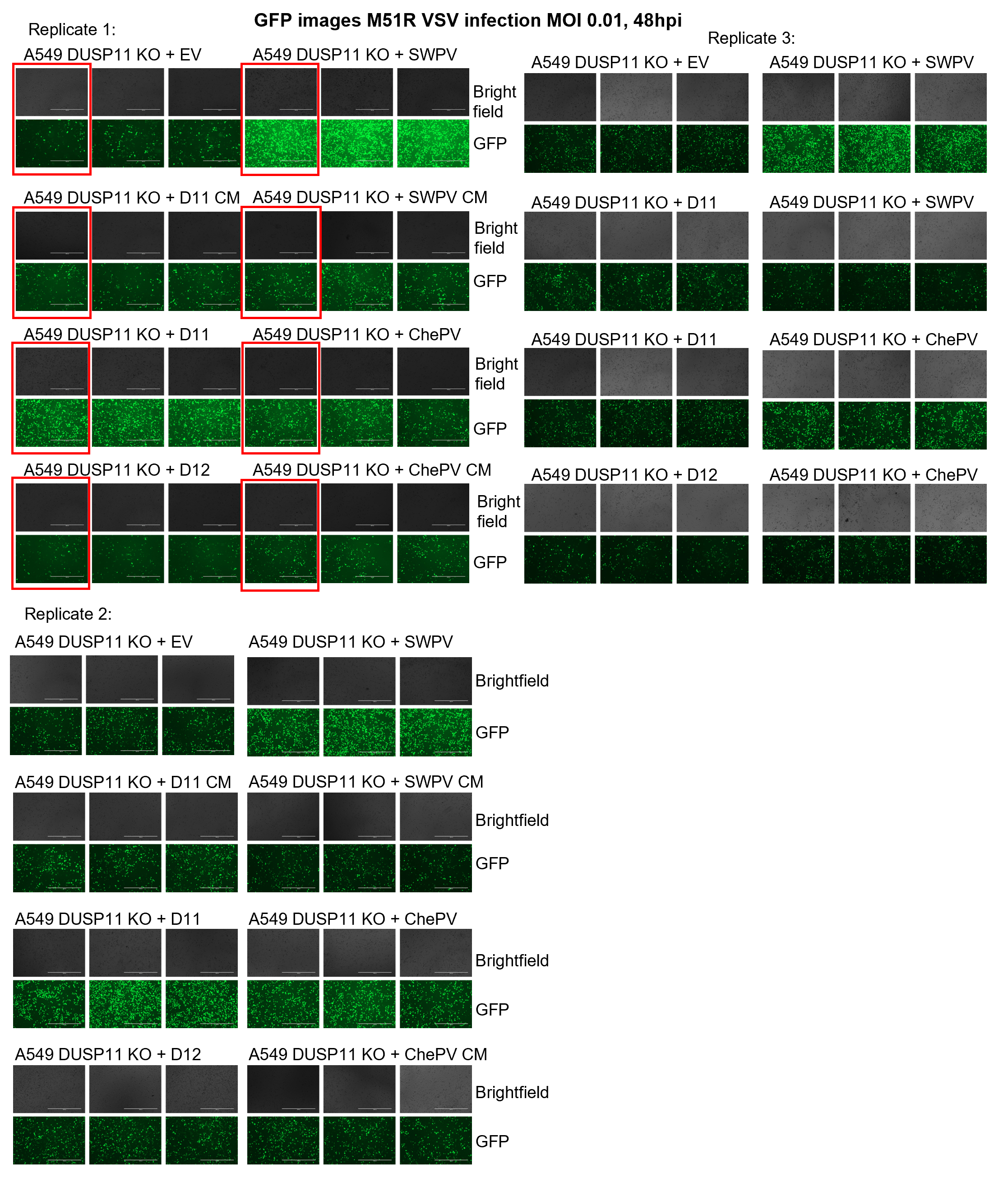

Supplement: S11 Fig — (TIF) [file ppat.1013101.s011.tif]

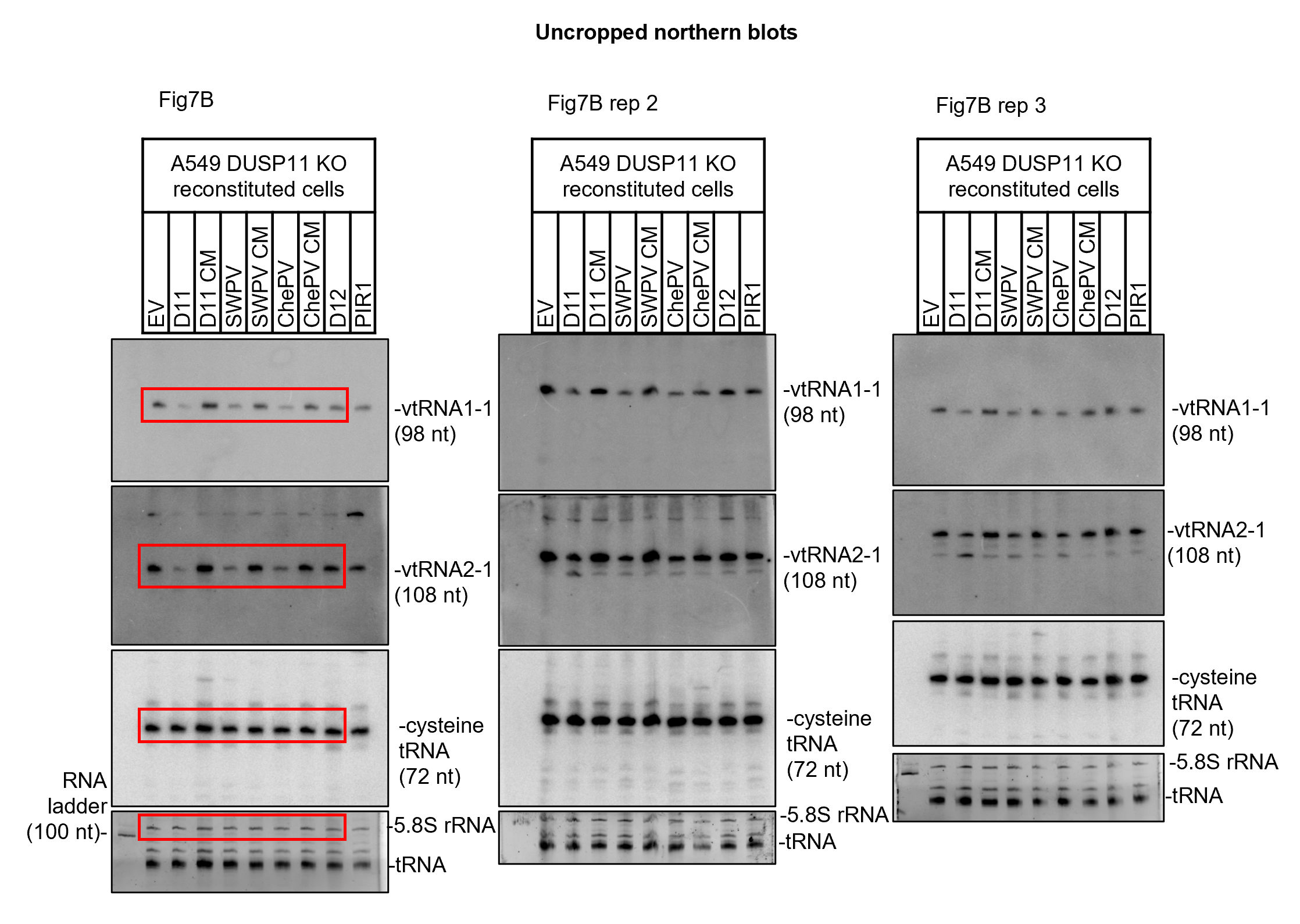

Supplement: S12 Fig — (TIF) [file ppat.1013101.s012.tif]

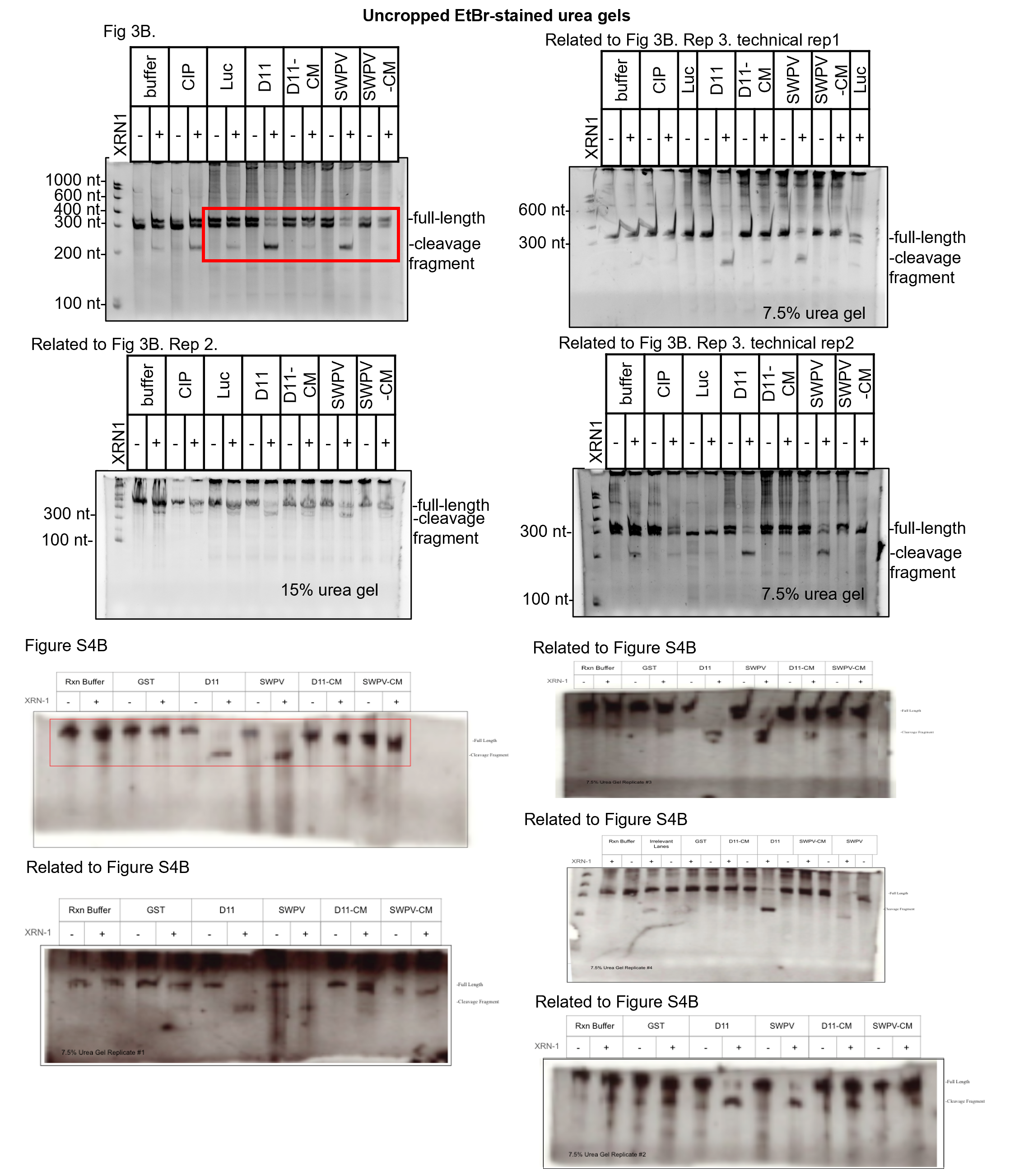

Supplement: S13 Fig — (TIF) [file ppat.1013101.s013.tif]

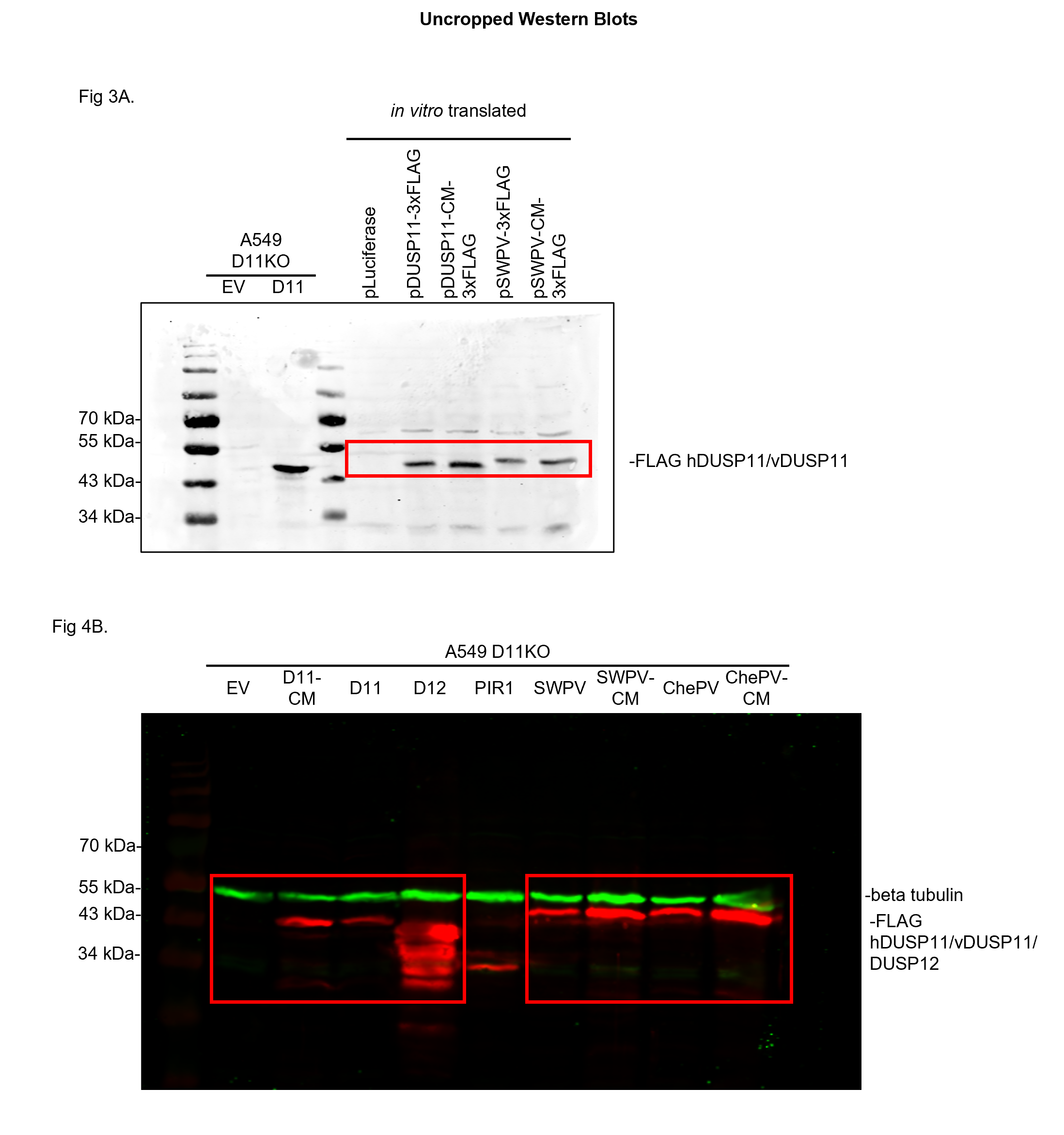

Supplement: S14 Fig — (TIF) [file ppat.1013101.s014.tif]
